# Meteorological change and hemorrhagic fever with renal syndrome epidemic in China, 2004–2018

**DOI:** 10.1038/s41598-022-23945-9

**Published:** 2022-11-21

**Authors:** Yizhe Luo, Heng Lv, Huacheng Yan, Changqiang Zhu, Lele Ai, Wenhao Li, Jing Yi, Lingling Zhang, Weilong Tan

**Affiliations:** 1 Department of Epidemiology, School of Public Health, Nanjing Medical University, Nanjing, 211166 China; 2 Nanjing Bioengineering (Gene) Technology Center for Medicines, Nanjing, 210002 China; 3 Department of Infectious Disease Control and Prevention, Center for Disease Control and Prevention of Southern Theatre Command, Guangzhou, 510507 China; 4 Department of Transfusion Medicine, Xijing Hospital, Fourth Military Medical University, Xi’an, 710032 Shaanxi China; 5 College of Life Science, Fujian Agriculture and Forestry University, Fuzhou, 350002 China

**Keywords:** Climate sciences, Ecology, Environmental sciences

## Abstract

Hemorrhagic fever with renal syndrome (HFRS), caused by *hantavirus*, is a serious public health problem in China. Despite intensive countermeasures including Patriotic Health Campaign, rodent control and vaccination in affected areas, HFRS is still a potential public health threat in China, with more than 10,000 new cases per year. Previous epidemiological evidence suggested that meteorological factors could influence HFRS incidence, but the studies were mainly limited to a specific city or region in China. This study aims to evaluate the association between monthly HFRS cases and meteorological change at the country level using a multivariate distributed lag nonlinear model (DLNM) from 2004 to 2018. The results from both univariate and multivariate models showed a non-linear cumulative relative risk relationship between meteorological factors (with a lag of 0–6 months) such as mean temperature (Tmean), precipitation, relative humidity (RH), sunshine hour (SH), wind speed (WS) and HFRS incidence. The risk for HFRS cases increased steeply as the Tmean between − 23 and 14.79 °C, SH between 179.4 and 278.4 h and RH remaining above 69% with 50–95 mm precipitation and 1.70–2.00 m/s WS. In conclusion, meteorological factors such as Tmean and RH showed delayed-effects on the increased risk of HFRS in the study and the lag varies across climate factors. Temperature with a lag of 6 months (RR = 3.05) and precipitation with a lag of 0 months (RR = 2.08) had the greatest impact on the incidence of HFRS.

## Introduction

HFRS is an emerging rodent-transmitted virus and global public health threat. The causative agents of HFRS include species of *hantaviruses* (HanVs), such as *Seoul virus*, *Hantaan virus*, *Puumala virus* and other viruses of the genus *Orthohantavirus*, mainly carried by rodents, insectivores and bats^1^. Transmission of *hantavirus* to humans occurred through contact with infected rodents, often through aerosolized urine and feces, causing *Hantavirus* Pulmonary Syndrome (HPS) in the Americas and HFRS in Europe and Asia^2^. With surge in globalization and the expansion of global trade and transports, rodent-borne *hantaviruses* have spread around the world. The epidemic HFRS in China, mainly caused by *Hantaan* virus and *Seoul virus*, accounts for 90% of global cases and is characterized by fever, hemorrhage, and acute kidney injury^3,4^. HFRS cases covered 31 provinces, municipalities and autonomous regions, with the average annual number of 10,000 HFRS cases in the past ten years^5^. After *hantavirus* was introduced into China, it selected suitable host animals here: a wide variety of rodents, shrews, bats, etc. Some studies explored that there are more than 8 suitable host species for *Seoul virus* and 10 species of wild mouse hosts for *Hantaan* virus in China^5,6^. A country with enormous variations in geographical span and a large range of climate types is suitable for survival and reproduction of host animals, making the HFRS difficult or impossible to eliminate^5,7,8^.

There are many potential risk factors for HFRS, which can be broadly classified as climate, socio-economic (e.g. gross domestic product (GDP), population density, food production), ecology (rodent population), rodent virus carrier rate, etc^9,10^. Meteorological factors, including temperature and humidity have delayed effects on the occurrence of HFRS, which may directly or indirectly influence the incidence by affecting the growth dynamics, activity frequency of rodents, and opportunity of virus-to-human contact^11^. Temperature, rainfall and relative humidity are three important factors that affecting HFRS cases^12,13^. For example, in Belgium, an outbreak of rodent-borne disease caused by *hantavirus*-infected bank voles (Myodes glareolus) was found to be positively associated with an increase in local mean temperature^14^. In North America, the increase in pasture yields after heavy rainfall in 1999 was associated with an increase in the Labrador white-footed rat (Peromyscus maniculatus) population, pushing the outbreak of HPS^15^. In northwestern Argentina, *hantavirus* transmission is positively correlated with lagging rainfall and temperature^16^. However, in the Weihe Plain in central China, the incidence of HFRS was negatively correlated with summer temperature, but positively correlated with summer precipitation^11^. Therefore, climatic variables may serve as indicators for the risk of human HFRS transmission. However, little is known about the effects of factors such as sunshine hour and wind speed on HFRS^17^. Sunshine hour and wind speed are thought to influence HFRS transmission by affecting factors such as crop yield, rodent reproduction and vector density. However, there is no direct evidence that they can be used as a risk indicator of HFRS transmission. Therefore, for the absence of an effective vaccine against HFRS, retrospective analysis of HFRS cases to speculate on potential risk factors will help the government to take targeted control measures more effectively.

In this study, we used the distributed lag nonlinear model (DLNM) to examine the nonlinear and distributed lag effects of temperature, precipitation, humidity, sunshine hour, and wind speed and to explore which factor is the best predictor of HFRS incidences using data from 31 provinces in China. To our knowledge, this is the largest HFRS epidemiological study to date, relying on the data from 31 provinces of China, covering the total population and the period between 2004 and 2018. This study aims to analyze the quantitative relationship between HFRS transmission and weather variables, predict the HFRS epidemics, and provide evidence for decision-making on strategies of HFRS prevention and control.

## Results

### HFRS distribution in China, 2004–2018

**Figure 1:**
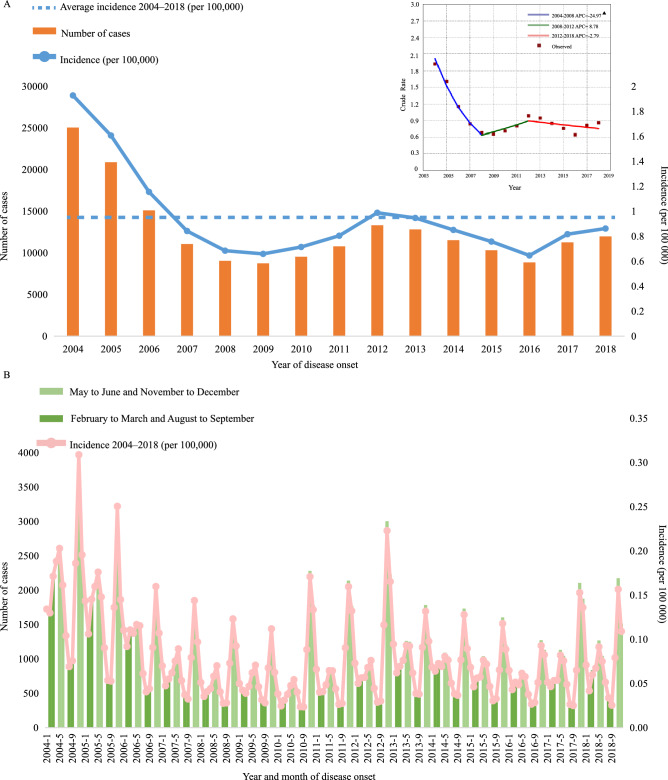
The incidence and number of HFRS cases reported in China, 2004–2018. (**A**) Number of cases and incidence by year. Trend of the incidence rate of HFRS between 2004 and 2018 shown by the joinpoint regression (upper right corner). The red squares represent the observed crude incidence of HFRS and the lines represent the slope of the annual percentage change (APC). (**B**) The pink line represents the monthly incidence of HFRS. The bar chart shows the number of cases at peak and trough.

From January 1, 2004 to December 31, 2018, 190 203 cases of HFRS were reported nationwide in China, with an average annual incidence rate of 0.950 per 100,000 people, with the highest incidence in 2004 (1.926 per 100,000) and the lowest in 2018 (0.86 per 100,000) (Fig. 1A), and the cases showed obvious seasonal fluctuations (Fig. 1B). HFRS cases existed every month and showed an obvious dual-season mode every year, with a spring peak from May to June and a winter peak from November to December. The highest number of cases were in May and November, with the composition ratios accounting of 9.51% and 17.06%, respectively (Fig. 1B).

The incidence of HFRS in northern regions was higher than that in the south, especially in Heilongjiang, Liaoning, Jining, Shaanxi, Shandong and Hebei provinces. Relatively few cases existed in south China, which were mainly concentrated in Jiangxi, Zhejiang, Hunan and Fujian (Figs. S1 and S2). Spatial autocorrelation analysis indicated that HFRS cases were positively correlated (Moran’s I = 0.09, p < 0.1), see Fig. S3. The incidence of HFRS noticeably decreased, with − 24.97% APC (95% confidence interval − 33.2 to − 15.7%, *P* = 0.001) before 2008, then remained stable until 2018 (*P* = 0.314, 2008–2012; *P* = 0.315, 2012–2018) (Fig. 1A). Obviously, residents can become infected with HFRS throughout the year.

We observed HFRS infection in all age groups, and the patients were mainly male, with a male to female ratio of 3:1. The age of onset was mainly between 35 and 75 years, with the highest annual mean incidence in the 50–55 age group (1.785 per 100,000; Fig. S4). The majority of HFRS cases were agricultural workers (121,777 cases, 68.32%), followed by domestic workers, housekeepers, the unemployed (21,147 cases, 11.8%), and industrial workers (20,574 cases, 11.54%) (Fig. S5).

### Meteorological factors distribution in China, 2004–2018

**Figure 2:**
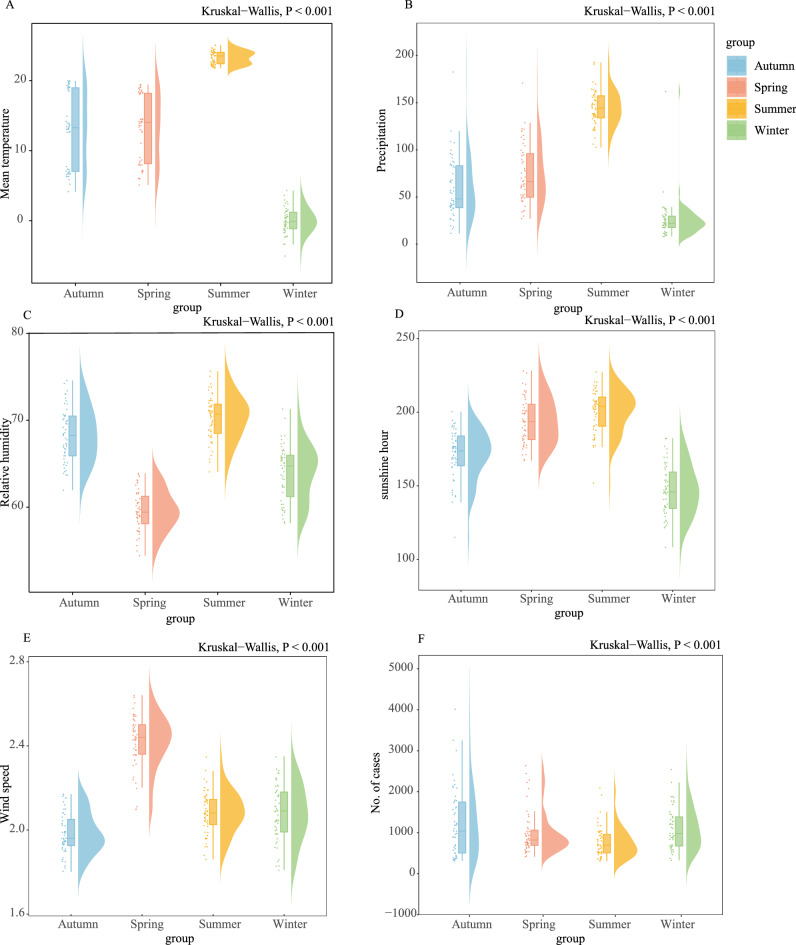
Boxplots of five meteorological variables and the number of HFRS cases in four seasons, 2004–2018 (n = 15*93 in each season). (**A**–**F**) Seasonal patterns of weather conditions. The *Kruskal–Wallis* test was used to compare the nonnormally distributed characteristics of five meteorological factors and HFRS incidence among the four seasons. Null hypothesis: the median values across the four seasons are equal. Alternative hypothesis: At least one of the median values of the four seasons is different from the others. Spring (March–May), summer (June–August), autumn (September–November) and winter (December–February).

The average annual Tmean was 13.27 °C, precipitation was 70.51 mm, RH was 66.22%, SH was 175.43 h, and WS was 2.15 m/s (Table 1). We also compared the mean values of each meteorological factor over the four seasons. Meteorological conditions show distinct seasonal changes, with higher Tmean, SH, RH and precipitation in summer, and higher WS in spring (Fig. 2).

**Table 1:** Descriptive statistics for monthly HFRS cases and weather conditions in China, 2004–2018.

Variables	Mean	SD	Min	P25	Median	P75	Max
Tmean (°C)	13.27	10.93	− 23.21	5.99	14.78	22.08	31.96
precipitation (mm)	70.51	63.91	0.00	15.21	50.16	113.82	200.00
RH (%)	66.22	13.15	27.43	57.33	69.06	76.93	89.33
SH (h)	175.43	57.94	11.43	137.17	179.33	217.72	325.45
WS (m/s)	2.15	0.47	0.79	1.79	2.12	2.50	3.00
No. of HFRS cases	34	75	0	1	9	38	1401

SD standard deviation; min. Minimum; P25 25th percentile; P50 median; P75 75th percentile, max. Maximum.

### Relationship between meteorological factors and HFRS, 2004–2018

**Figure 3:**
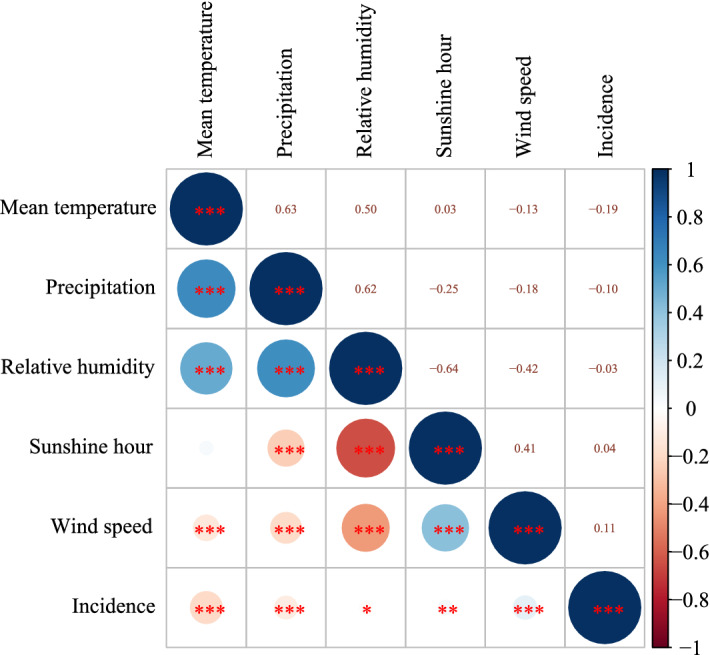
Pearson correlation coefficient between weather conditions and HFRS in China. *0.05 ≥ p > 0.01; **0.01 ≥ p > 0.001; ***≤ 0.001.

Pearson's correlation analysis revealed that the incidence of HFRS was correlated with meteorological factors: WS (r = 0.11***), SH (r = 0.04**), Tmean (r = − 0.19***), precipitation (r = − 0.1***), and RH (r = − 0.03*) (Fig. 3).

**Figure 4:**
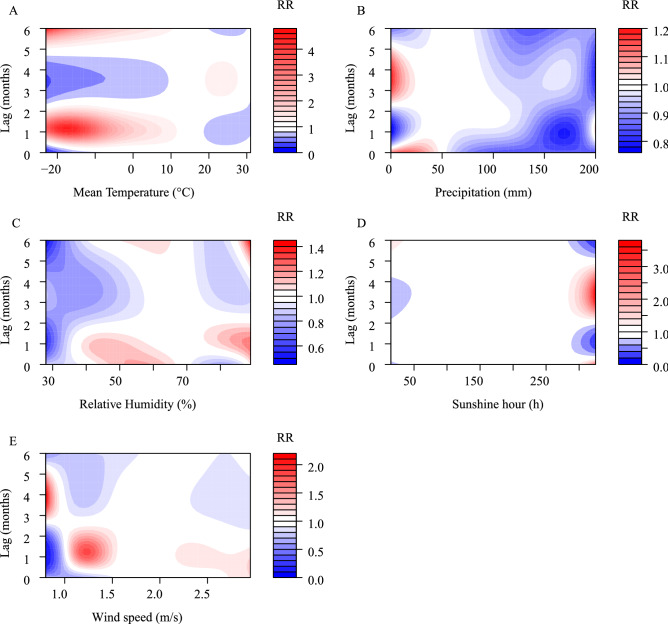
Contour plot of the exposure–response relationship between the incidence of HFRS and five meteorological conditions in the univariate model. The Y-axis represents the lag period from 0 to 6 months. The x-axis represents the range of observations for each variable. RR stands for relative risk, red stands for RR > 1, white stands for RR = 1, and blue stands for RR < 1.

The DLNM model showed an association between HFRS and the five meteorological conditions, with a lag of 0–6 months. In the univariate models (Fig. 4), five meteorological conditions were associated with HFRS incidence, with relative risks (RR) ranging from 0.17 to 4.68 at Tmean, 0.76–1.19 for precipitation, 0.48–1.44 for RH, 0.20–3.73 for SH, and 0.08–2.20 for WS. The maximum RR values, including commensurable meteorological and lag time, for the five meteorological conditions were 4.68 (Tmean, − 23 °C, lag 6 months), 1.13 (precipitation, 0 mm, lag 3.6 months), 1.44 (RH, 89%, lag 6 months), 3.73 (SH, 325 h, lag 3.4 months), 2.20 (WS, 0.8 m/s, lag 3.8 months), respectively.

**Figure 5:**
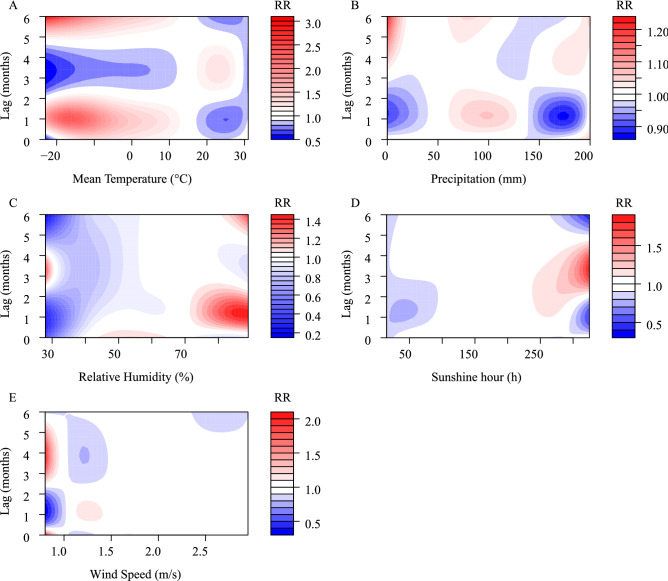
Contour plot of the exposure–response relationship between the incidence of HFRS and five meteorological conditions in the multivariate model. The Y-axis represents the lag period from 0 to 6 months. The x-axis represents the range of observations for each variable. RR stands for relative risk, red stands for RR > 1, white stands for RR = 1, and blue stands for RR < 1.

In the multivariate models (Fig. 5), the RR values were 0.52–3.05 (Tmean), 0.87–1.22 (precipitation), 0.20–1.44 (RH), 0.39–1.86 (SH), 0.32–2.08 (WS), respectively. The maximum RR values, including commensurable meteorological and lag time, for the five meteorological conditions were 3.05 (Tmean, − 23 °C, lag 6 months), 1.22 (precipitation, 0 mm, lag 6 months), 1.44 (RH, 87%, lag 1.2 months), 1.86 (SH, 325 h, lag 3.4 months), 2.08 (WS, 0.8 m/s, lag 0 months), respectively.

### Cumulative relative risks at 0–6 months lag

**Figure 6:**
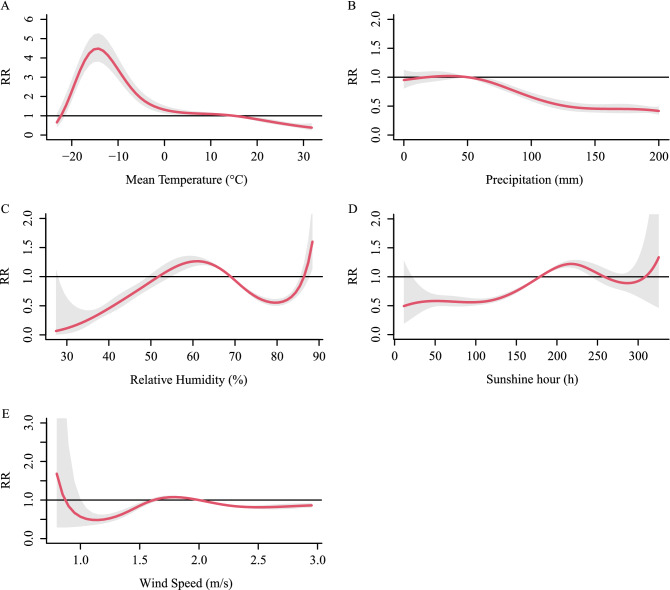
Summary of cumulative exposure–response curves for HFRS incidence with a lag of 0–6 months for meteorological factors from 2004 to 2018 in the univariate model. The Y-axis represents the relative risk of each variable. The x-axis represents the range of observations for each variable. Red lines represent means estimated using the DLNM model, shaded areas represent 95% confidence intervals.

**Figure 7:**
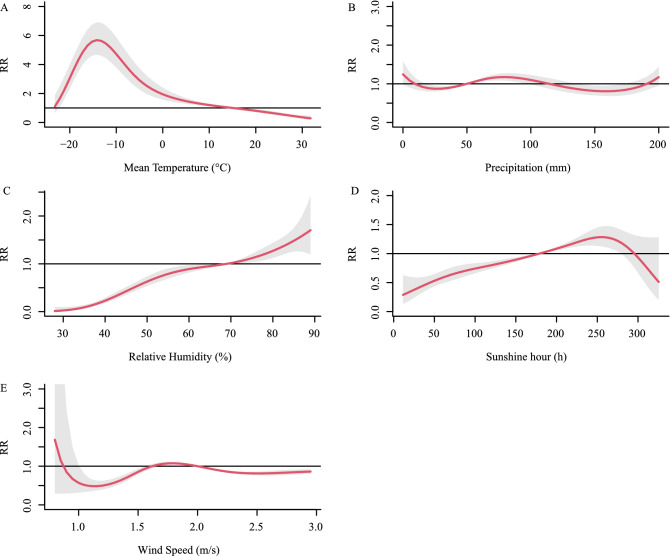
Summary of cumulative exposure–response curves for HFRS incidence with a lag of 0–6 months for meteorological factors from 2004 to 2018 in the multivariate model. The Y-axis represents the relative risk of each variable. The x-axis represents the range of observations for each variable. Red lines represent means estimated using the DLNM model, shaded areas represent 95% confidence intervals.

We found that the cumulative relative risk of meteorological factors (with 0–6 months lag) was associated with HFRS incidence. In the univariate models (Fig. 6), the meteorological conditions associated positively with HFRS risk are -22.2–14.8 °C (Tmean), 55.4–68.4% (RH) & 86.4–88.4% (RH), 18–50 mm (precipitation), 179.4–258.4 h (SH), 2.00–2.15 m/s (WS). In the multivariate models (Fig. 7), the meteorological conditions are -23–14.79 °C (Tmean), 69–89% (RH), 50–95 mm (precipitation), 179.4–278.4 h (SH), 1.70–2.00 m/s (WS).

### The lag relationship between meteorological factors and the incidence of HFRS

The results of DLNMs were shown in Fig. S6. In multivariate models, median values of Tmean, precipitation, RH, median SH and WS being used as references, the RR of HFRS incidence with lag 0–6 months were calculated with the 2.5th, 25th, 75th and 97.5th percentile of Tmean, precipitation, RH, SH and WS, respectively. The multivariate plots showed that RR was significantly high (RR > 1) from lag month 0 (RR = 1.31, 95% CI 1.06–1.62) to lag month 2.2 (RR = 1.11, 95% CI 1.01–1.22). Under the 25th Tmean, high RRs were observed from lag month 0 (RR = 1.13, 95% CI 1.03–1.24) to lag month 1.8 (RR = 1.09, 95% CI 1.03–1.16) under the extremely low Tmean (2.5th percentile). Under the 75th RH, the RRs were significantly high for a lag of 0.4 months (RR = 1.03, 95% CI 1.00–1.07) to 2.6 months (RR = 1.05, 95% CI 1.02–1.08). Furthermore, the RRs for lag month 0.4 (RR = 1.09, 95% CI 1.03–1.16) to month 2.4 (RR = 1.09, 95% CI 1.04–1.14) and lag month 5.6 (RR = 1.07, 95% CI 1.01–1.13) to lag month 6 (RR = 1.13, 95% CI 1.05–1.22) were significantly high under the 97.5th percentile of RH. Under the 75th SH, a lag of 1.6 months (RR = 1.03, 95% CI 1.01–1.06) to a lag of 5.2 months (RR = 1.02, 95% CI 1.01–1.04) RRs were high.

Figure S7 showed the lag-specific association between meteorological factors and HFRS incidence. Significant RRs were observed at lags of 3 and 6 months when Tmean was 15–27 °C and -23–15 °C, respectively. Precipitation of 0–15 and 55–90 mm resulted in significantly higher RR values after 6 months. When RH exceeded 82% after 6 months, the RR was significantly high. In addition, SH and WS with a lag of 3 months had high RRs at 180–325 h and 2.55–2.75 m/s, respectively.

## Discussion

Based on the data from the National Notifiable Communicable Disease Surveillance System and the meteorological monitoring database spanning 2004–2018 years, the incidence rates of HFRS were generally declining and showed spatial aggregation with most cases distributed in the three northeastern provinces and eastern coastal provinces. The majority of patients are from male agricultural workers. HFRS incidences showed a bimodal pattern every year, with the monthly incidence lower in summer and faster in spring and winter. This study explored the delayed effect of meteorological factors on the HFRS epidemic in China. Our results showed that mean temperature, precipitation, relative humidity, sunshine hour, and wind speed have different degrees of delayed effects on the occurrence of HFRS.

Quite a few studies have shown that economic development (such as urbanization, population migration, etc.) always improve the human living environment and enhance self-protection awareness, at the same time, it can also change the rodent community structure, population number and habitat, resulting in changes in the mechanism of HFRS^18^. The results of this study showed that a lower level of economic development in northeast China co-existed with the highest incidence of HFRS, which indicated that economic development level and urbanization rate were indeed related to the incidence of HFRS besides meteorological factors^17^. However, rodent densities in less developed areas are higher than those in metropolitan areas^17^. These results emphasized that economic and social development could reduce the transmission of HFRS by reducing rodent density^17^. Urbanization and large-scale construction will directly or indirectly affect rodent living environment and foraging, resulting in the change of disease transmission intensity^19^. Agricultural workers are the high-risk group for contracting HFRS and the reasons may include Chinese large population of agricultural workers, their manual labor modes and work locations, which were adjacent to residential areas and suitable for rodent survival and reproduction^12^.

Meteorological factors may directly or indirectly influence the occurrence of HFRS, mainly by affecting the infection rate and population dynamics of the host (reproduction and activity of rodents), the regeneration of mites, and the rate of rodent-human contact. Vector-borne viral diseases, including HFRS, are among the most climate-sensitive diseases of all^20^.

DLNM was used to explore the monthly lag effects of different climate factors on HFRS, and the lag effects vary across climatic factors was uncovered. The different lag periods suggested that the delayed effects of each climate variable may be related to the spread of HFRS infection being influenced by various factors, including the density of host rodents, hantavirus positivity, and frequency of exposure to humans, etc^21,22^. DLNM, by Sun et al. in Huludao City, Northeast China, conducted and identified extremely high temperature with a lag of 15–16 weeks, extremely low temperature with a lag of 5–6 weeks, extremely high humidity with a 10–11 weeks lag being strongly associated with HFRS. It also identified the 5-week lag with temperature of − 8–10 °C and the 15-week lag with temperature exceeded 23 °C would led to the significantly high RR of HFRS^13^. The highest temperature of the year occuring between August and September, while the HFRS cases increasing to a peak in November and December, thus indicated that the incidence of HFRS may lag behind temperature by about 3 months. Our results fitted this evidence for it ^23^.

A previous study showed that temperature could influence rodent abundance, frequency of human-rodent contact and *hantavirus* disease risk and affect vegetation growth, reproduction and survival of rodent^13^. These factors are related to the survival time of infectious *hantavirus* in the environment^24^. In this study, both univariate and multivariate models showed that there was a significant nonlinear effect between the incidence of HFRS and the monthly average temperature. Overall, results exhibited a negative correlation, which was consistent with previous discoveries^11^. The reason may be that lower temperature may affect rodent reproduction rate, litter size and survival rate. Furthermore, low temperature prolong the survival time of virus outside the host, even in the absence of direct rodent contact or rodent-to-human contact, the host virus remains infectious^25^. In addition, significant RR were observed with a 3-month lag at 15–27 °C indicated a positive correlation between HFRS and temperature, illustrating that temperature affects the incidence of HFRS in multiple ways and may be affected by the environment, climate, seasonal changes in rodent populations, and viral spread ^26^.

Opposite results were discovered in both univariate and multivariate models, possibly because climate variables such as temperature and RH may have an interaction effect on precipitation, which were not shown in the univariate model^13^. Sun et al. found an interaction effect between temperature, RH, and precipitation on HFRS incidence. Increasing precipitation and rising temperatures (< 14.79 °C) can collectively boost the risk of HFRS infection, which was similar to our results ^13^. However, excessive precipitation may be a risk factor for HFRS transmission, which can damage their nests, making them difficult to obtain food ^27^.

Our study showed that high relative humidity was significantly associated with the incidence of HFRS. A humid environment and high relative humidity were conducive to the survival or reproduction of mites^28^. Humidity may affect the *hantavirus* positivity rate in rodents by promoting mites reproduction and infestation, ultimately increasing the risk of HFRS^28^.In a study of the association of meteorological factors with HFRS in 19 cities, the risk of scrub typhus increased by 0.9% (95% CI 0.5–1.2%) for every 1% increase in monthly relative humidity; with a lag of 16 weeks^29^. In another Belgian study, high humidity was described as critical for *Puumala hantavirus* (PUUV) survival, and humid conditions were thought to favor PUUV transmission among rodents^30^.

Several empirical studies have confirmed that long duration of solar radiation have a positive effect on the incidence of infectious diseases such as mumps (Jining, Shandong Province) and scarlet fever (Beijing), and these findings are consistent with our conclusions^31–33^. Similar to previous studies, our study also found an increased risk of HFRS when the monthly mean WS was favorable^34^. The possible reason was that wind could resuspend bacteria or virus and increase the concentration and survival time of virus. On the other hand, wind might promote the transmission of *hantavirus* by increasing air flow. Sunshine hour and wind speed could have an impact on crop yield, rodent reproduction and vector density, which may affect the probability of HFRS occurrence ^35,36^.

In conclusion, meteorological factors have delayed effects on the HFRS incidence and their lag effects are not completely consistent with each other. We should pay more attention to HFRS control according to the weather conditions with less precipitation and 3 months after the temperature of 15–27 °C, sunshine hour of 180–325 h, and wind speed of 2.55–2.75 m/s and consider the lag response. This study also has several limitations. First of all, we could not calculate the age-standardized incidence of HFRS because of the lack of age-specific case information. Secondly, we unable to reflect the differential association between meteorological factors and HFRS risk in different regions. Moreover, we were unable to calculate the acute impact of meteorological factors on HFRS from monthly data. Finaly, some risk factor data were not collected, including land cover, pathogen dynamics, rodent density, and animal infection rates. This study investigated the relationship between meteorological conditions and the incidence of HFRS in China through the time lag effect. It is necessary to comprehensively consider the impact of pathogenic factors and socioeconomic environment on individuals in the future.

## Methods

### Data source and collection of case data

The monthly meteorological data of 839 stations in 31 provinces were collected from the National Meteorological Information Center (http://data.cma.cn/wa), including Tmean, precipitation, RH, SH, and WS (see Fig. S8). HFRS is a class B notifiable infectious disease in China^37^ and were diagnosed according to China’s Diagnostic Criteria for Epidemic Hemorrhagic Fever issued by the Ministry of Health (http://www.nhc.gov.cn/wjw/s9491/200802/39043.shtml). HFRS case-level records from 2004 to 2018 for our study were obtained from the Notifiable Infectious Disease Surveillance System (NIDSS) and sorted by province and month.

### Statistical analysis

Incidence (per 100,000 people) was defined as the number of HFRS cases per year divided by population size. The Joinpoint Regression Program (version 4.5.0.1), developed by the National Cancer Institute (NCI), was used to calculate the annual percentage change of crude incidence rates for 2004–2018. A *two-tailed t-test* was used to assess whether the annual percentage change in incidence rates was significantly different from 0. The χ^2^ test was used to compare the proportions of patients by sex, age, and occupation. In descriptive analyses, mean, standard deviation, minimum and maximum and quartiles (P_25_, P_50_, P_75_) were used to describe the distribution of HFRS incidence and meteorological variables. The level was set at 0.05. All maps were created using ArcGIS 10.2 (Esri Inc, Redlands, CA, USA) (http://desktop.arcgis.com).

### DLNM models

DLNM represented a modeling framework that could flexibly describe associations to show potential nonlinear and lagged effects in time series data. Here, we used the DLNM model to assess the impact of meteorological factors on HFRS incidence. A Pearson correlation analysis was used to analyze the relationship between HFRS incidence and climatic factors. In addition, the absolute value of the *pearson* correlation coefficient ≥ 0.7 was considered a strong correlation and would not be included in the DLNM model at the same time. Previous study found that monthly HFRS case was count data, which was a small probability event and obeyed a Poisson distribution. Taking the monthly cases of HFRS as the dependent variable, we established a crossbasis for five different meteorological factors (Tmean, precipitation, RH, SH, and WS), and incorporated the monthly meteorological data into the model in the form of crossbasis, while controlling the influence of long-term trends, seasonality and other confounding factors. We specified the crossbasis matrix by the function B-splines. Based on previous research, QBIC and experience, we set the maximum time lag at 6 months^38^. In this study, the median of each variable was selected as the reference value. In order to analyze the long-term effects of various meteorological factors on the incidence of HFRS, the establishment of the DLNM model was carried out in the following two stages.

In the first stage, a univariate DLNM model of five meteorological factors was established. We considered not only meteorological factors in the univariate model, but also long-term time trends, quartiles of mean incidence in 31 provinces, and incidence in the previous month. According to the principle of the smallest AIC value of the model, we determined the optimal model:

$$\begin{aligned} {\text{Model}}  = {\text{glm}}\;({\text{case}}\sim {\text{cb}}1.{\text{X}} + {\text{ns}}\;({\text{seq}},\;15*7) + {\text{factor}}\,({\text{season}}) + {\text{factor}}\,({\text{region}}) + {\text{lag}}.{\text{value}}1,\,{\text{family}} = {\text{quasipoisson}}(),\,{\text{new}}\_{\text{ds}}). \\ \end{aligned}$$

“case” represented the number of monthly counts of HFRS cases; “cb1.X” represented the cross-basis for five meteorological factors; “X” represented one of the five meteorological factors (Tmean, precipitation, RH, SH, and WS); “seq” represented the long-term trend, “15” represented years 2004–2018, “7” represented the degrees of freedom per year were used; “season” and “region” represented the covariate; “lag.value1” represented mean incidence in the previous month; “new_ds” represented our dataset.

In order to comprehensively consider the impact of the five meteorological factors on the incidence of HFRS. In the second stage, a multivariate DLNM model of five meteorological factors was established. We considered not only five meteorological factors (Tmean, precipitation, RH, SH, and WS), but also long-term time trends, seasonal changes (A year was divided into four seasons: spring (March to May), summer (June to August), autumn (September to November), and winter (December to February)), and incidence rates over the past month. According to the principle of the smallest AIC value of the model, we determined the optimal model:

$$\begin{aligned} {\text{Model}} = {\text{glm}}\;({\text{case}}\sim {\text{cb}}1.{\text{prec}} + {\text{cb}}1.{\text{rh}} + {\text{cb}}1.{\text{temp}} + {\text{cb}}1.{\text{wind}} + {\text{cb}}1.{\text{sun}} + {\text{ns}}\;{\text{(seq}},\;15*7{)} + {\text{factor}}\;{\text{(season)}} + {\text{factor}}\;({\text{region}}) + {\text{lag}}.{\text{value}}1,\;{\text{family}} = {\text{quasipoisson}}(),\;{\text{new}}\_{\text{ds}}). \\ \end{aligned}$$

“case” represented the number of monthly counts of HFRS cases; “cb1. prec” represented the cross-basis for precipitation; “cb1. rh” represented the cross-basis for RH; “cb1. temp” represented the cross-basis for Tmean; “cb1. wind” represented the cross-basis for WS; “cb1. sun” represented the cross-basis for SH; “seq” represented the long-term trend, “15” represented years 2004–2018, “7” represented the degrees of freedom per year were used; “season” and “region” represented the covariate; “lag.value1” represented mean incidence in the previous month; “new_ds” represented our dataset. All analyses in our study were performed using the package “dlnm” (version 4.1.3, https://cran.r-project.org/web/package/dlnm/index.html) in R software (version 4.1.3). The contour plot and exposure-effect curve of climate variables at different lag times were made to clarify the lag effect and its duration under different meteorological conditions. Taking the median of different meteorological conditions as a reference, the relationship between P2.5, P25, P75, P97.5 of the modified meteorological conditions and the incidence of HFRS was calculated respectively. The relative risk (RR) was used to assess the impact of meteorological factors with different lag times on the incidence of HFRS.

#### Sensitivity analysis

We rely on QBIC to select the optimal number and location of nodes reduced to determine natural splines. The final model should have the smallest sum of QBICs for all 31 provinces. We used AIC to select degree time variables, including a natural cubic spline of elapsed time, with seven degree of freedom (df = 7) per year, to control for long-term trends in meteorological variables in each province.

## Supplementary Information

Supplementary Information.

## Data Availability

In this paper, we used the meteorological data from the National Meteorological Information Center (http://data.cma.cn/wa) and HFRS cases data from the Chinese Center for Disease Control and Prevention (CDC) (http://www.chinacdc.cn/).

## Abbreviations

HFRS: Hemorrhagic fever with renal syndrome
DLNM: Distributed lag nonlinear model
Tmean: Mean temperature
RH: Relative humidity
SH: Sunshine hour
WS: Wind speed
PUUV: Puumala hantavirus
NCI: National Cancer Institute
