# Supplementary material for: Meteorological change and hemorrhagic fever with renal syndrome epidemic in China, 2004–2018
Source: Sci Rep. 2022 Nov 21;12:20037. doi: 10.1038/s41598-022-23945-9 (PMC9681842; doi:10.1038/s41598-022-23945-9)
Supplement: Supplementary file 1 — Supplementary Information. [file 41598_2022_23945_MOESM1_ESM.docx]

**Association between meteorological change and hemorrhagic fever with renal syndrome（HFRS）epidemic in China: a 15-year surveillance study**

**Fig. S1: Spatiotemporal distribution of HFRS cases in China, 2004-2018**

**
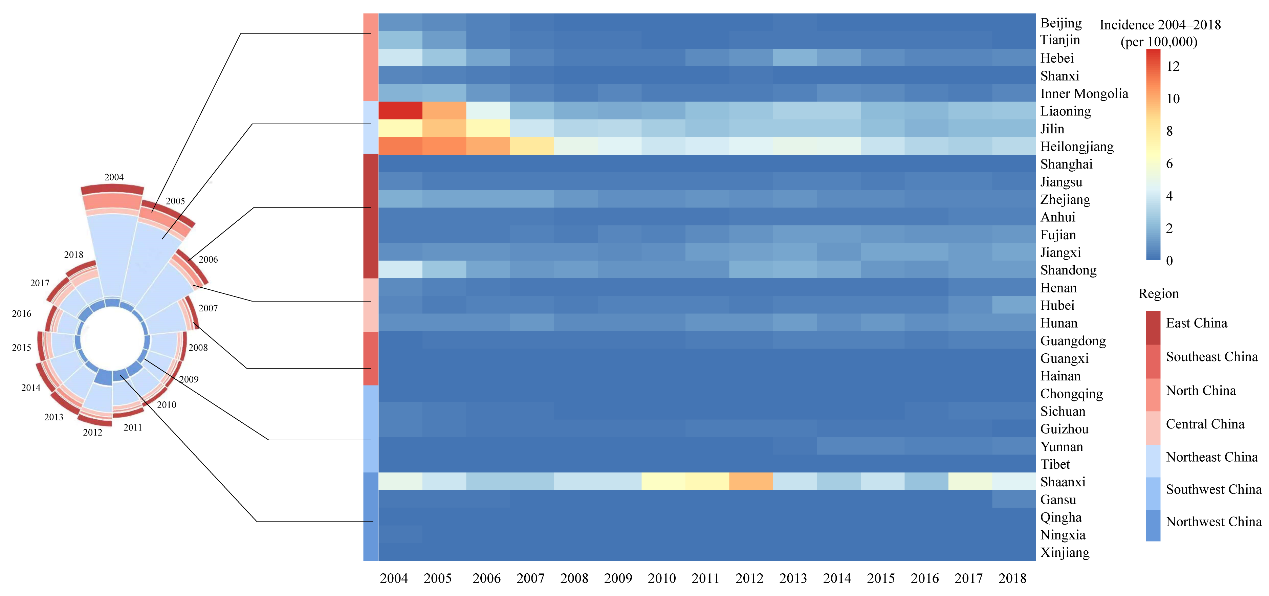
**

Illustration of seven geographical regions of China with their respective incidence, using circular bar plots (“rose plots”). Heatmap shows the annual incidence of pertussis per 100,000 people in 31 provinces of China (Heatmaps).

**Fig. S2: Spatiotemporal and average temperature distribution of HFRS cases in China, 2004-2018**

**
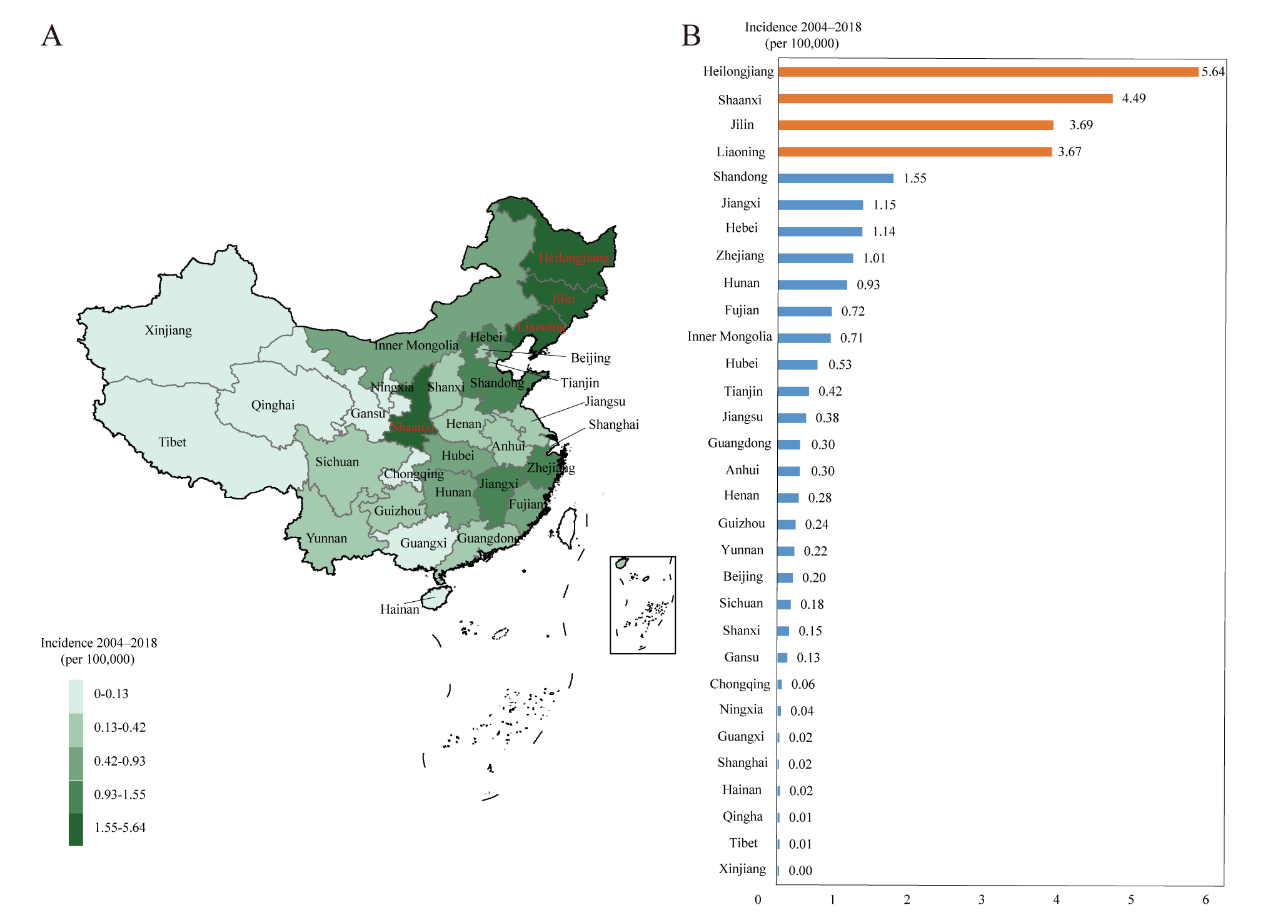
**

(A) The map represents the average incidence of HFRS in 31 provinces in China from 2004 to 2018. (B) The bar chart shows the average incidence by province from 2004 to 2018, with the top four provinces with the highest incidence indicated in orange.

The map was created using ArcGIS 10.2 (Esri Inc, Redlands, CA, USA) (http://desktop.arcgis.com).

**Fig. S3: Spatial autocorrelation analysis of HFRS incidence in China, 2014 to 2018**

**
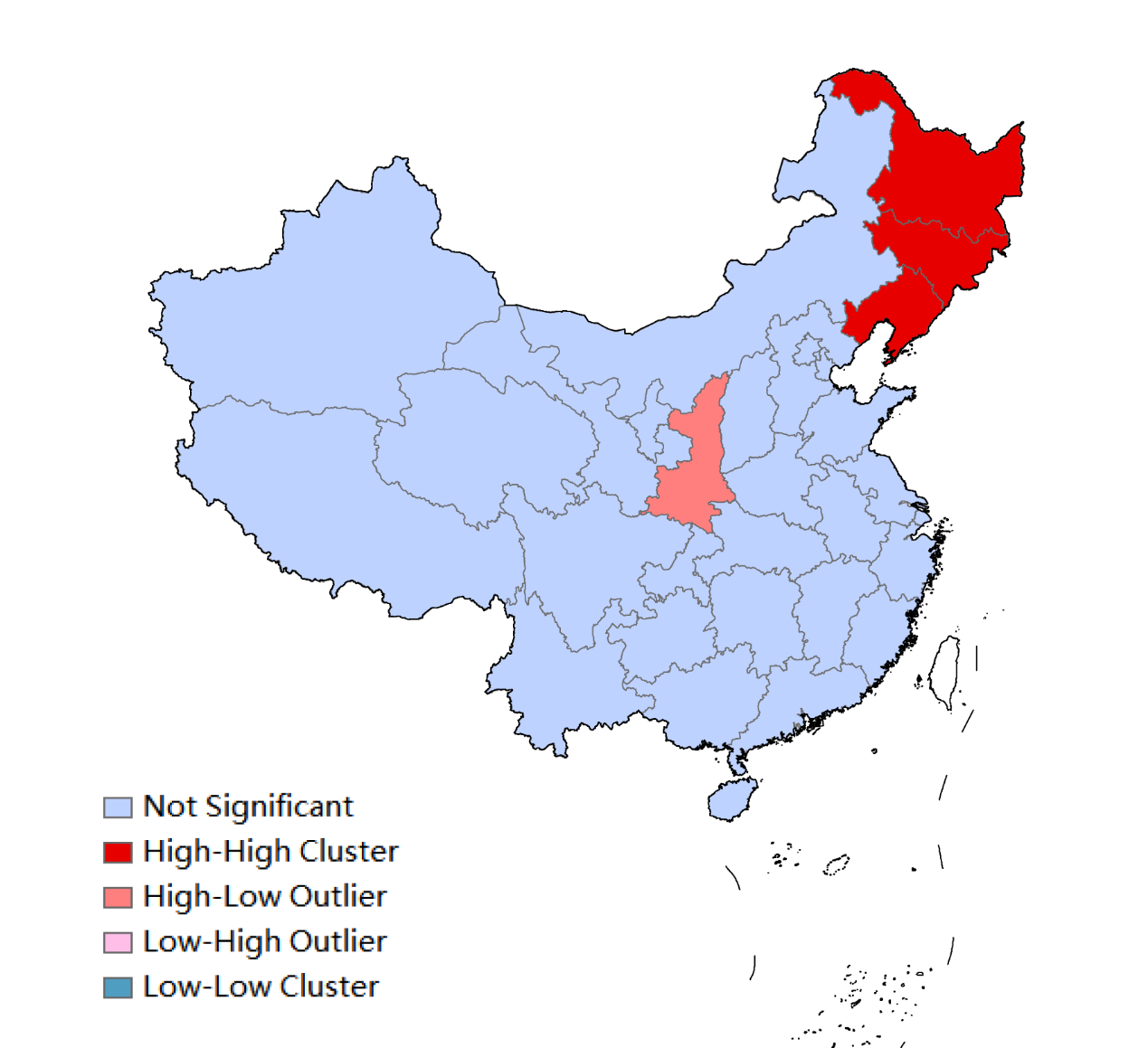
** The map was created using ArcGIS 10.2 (Esri Inc, Redlands, CA, USA) (http://desktop.arcgis.com).

**Fig. S4: Sex-specific distribution of HFRS incidence by age in China, 2004-2018**


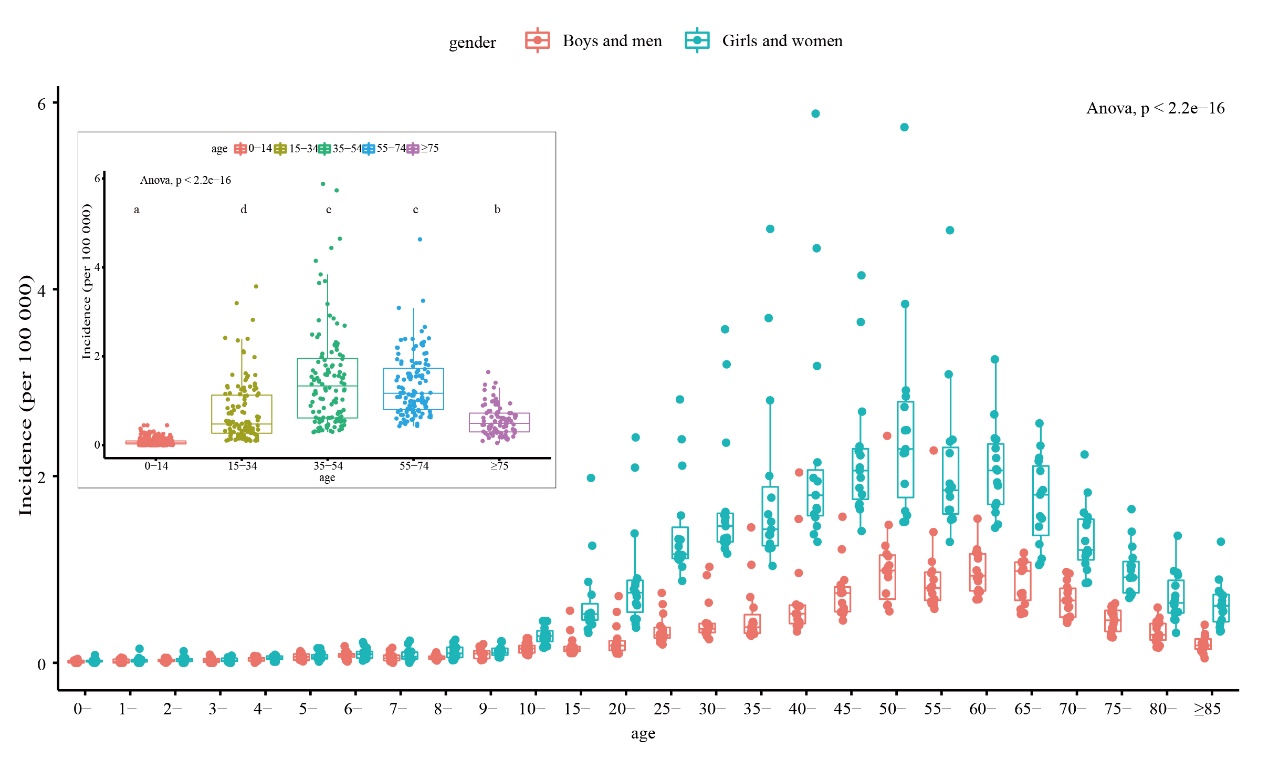


Boxes represent 50% of the cases distribution and the line indicates the median of the data.

**Fig. S5: Occupational distribution of HFRS cases in China, 2004-2017**

**
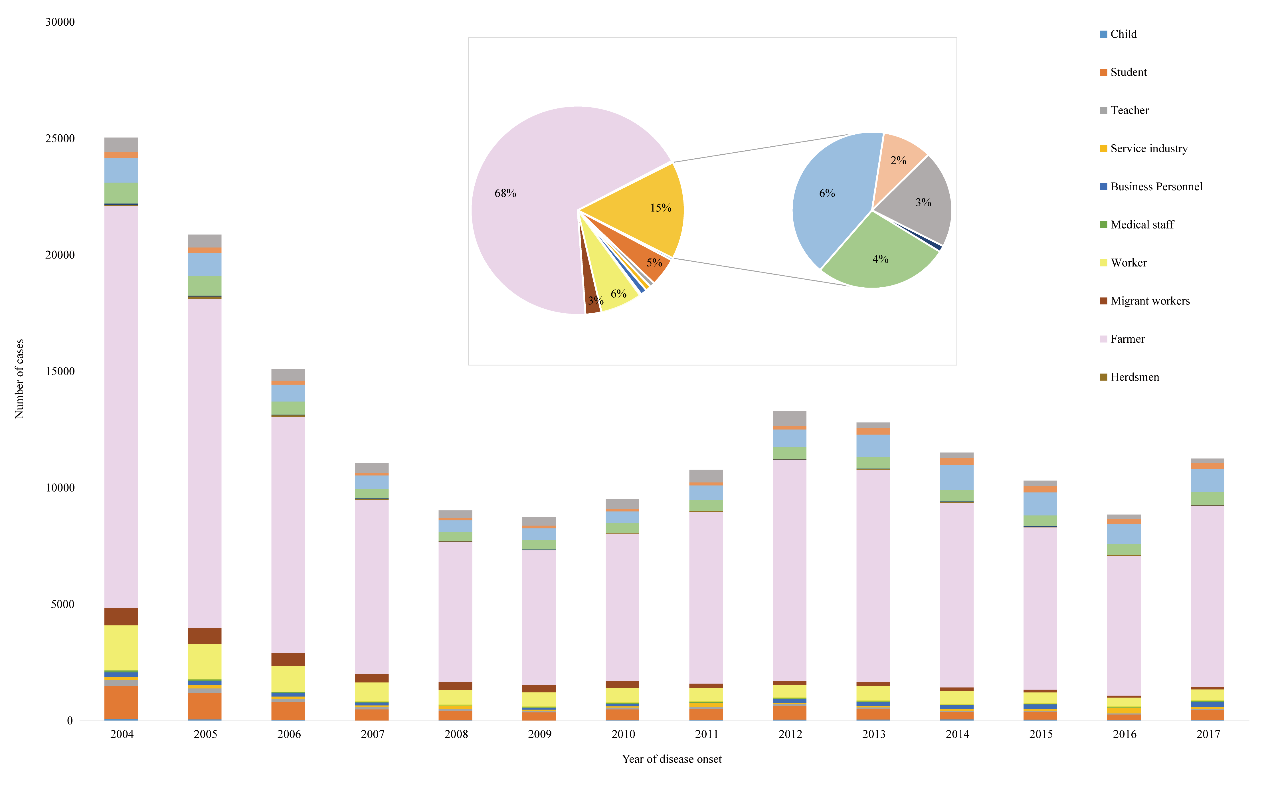
**

The stacked bar chart shows the changes in occupational distribution of HRFS patients from year to year. Left pie chart shows the proportion of patients in different occupations; right pie chart shows the percentage of occupations with low incidence.

**Fig. S6: Summary of** **relative risk curves for different lags between meteorological factors and HFRS incidence in** **multivariate models from 2004 to 2018**


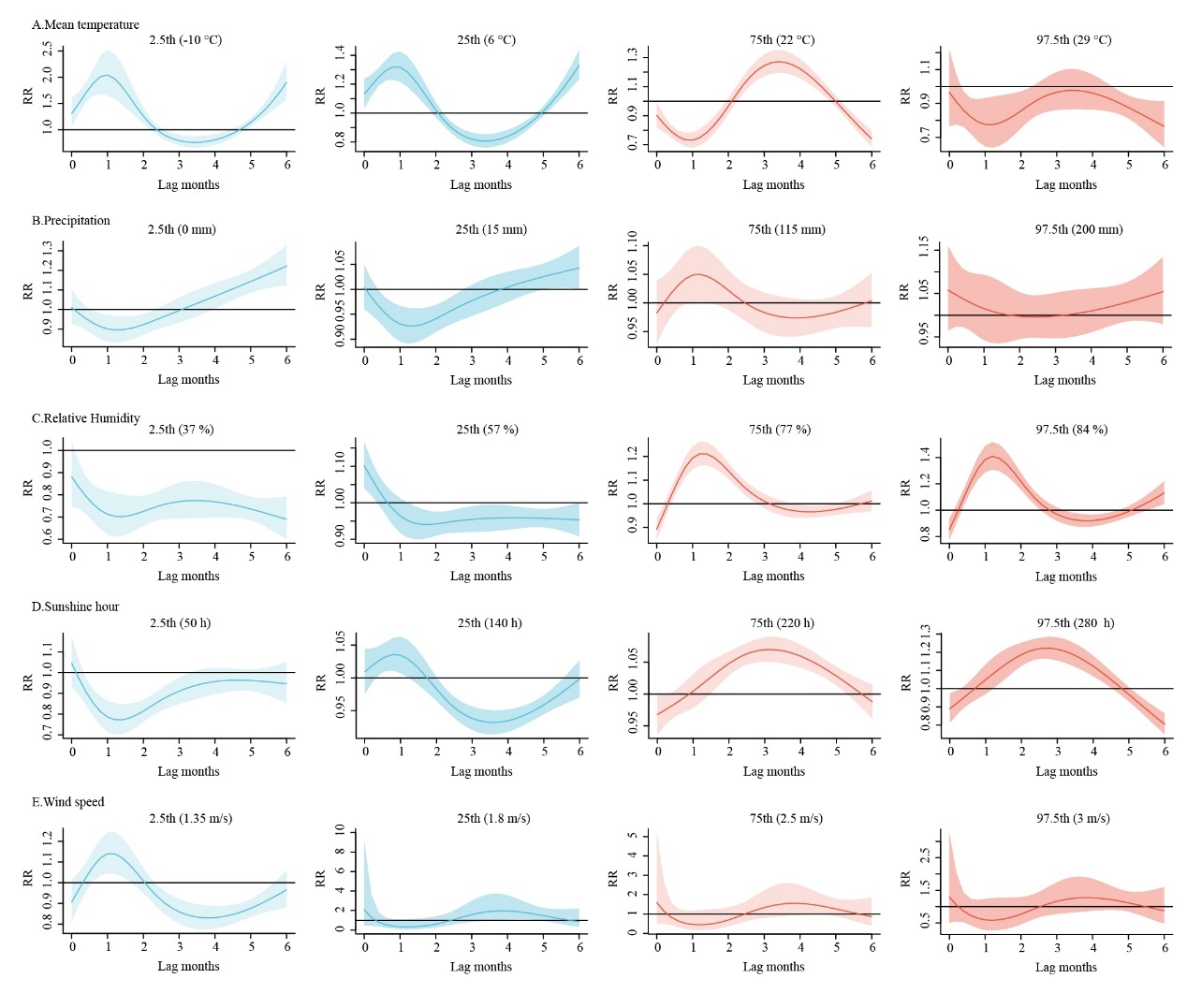


The Y-axis represents the relative risk of each variable, the x-axis represents the lag period from 0 to 6 months. Lines represent means estimated using the DLNM multivariate model, shaded areas represent 95% confidence intervals.


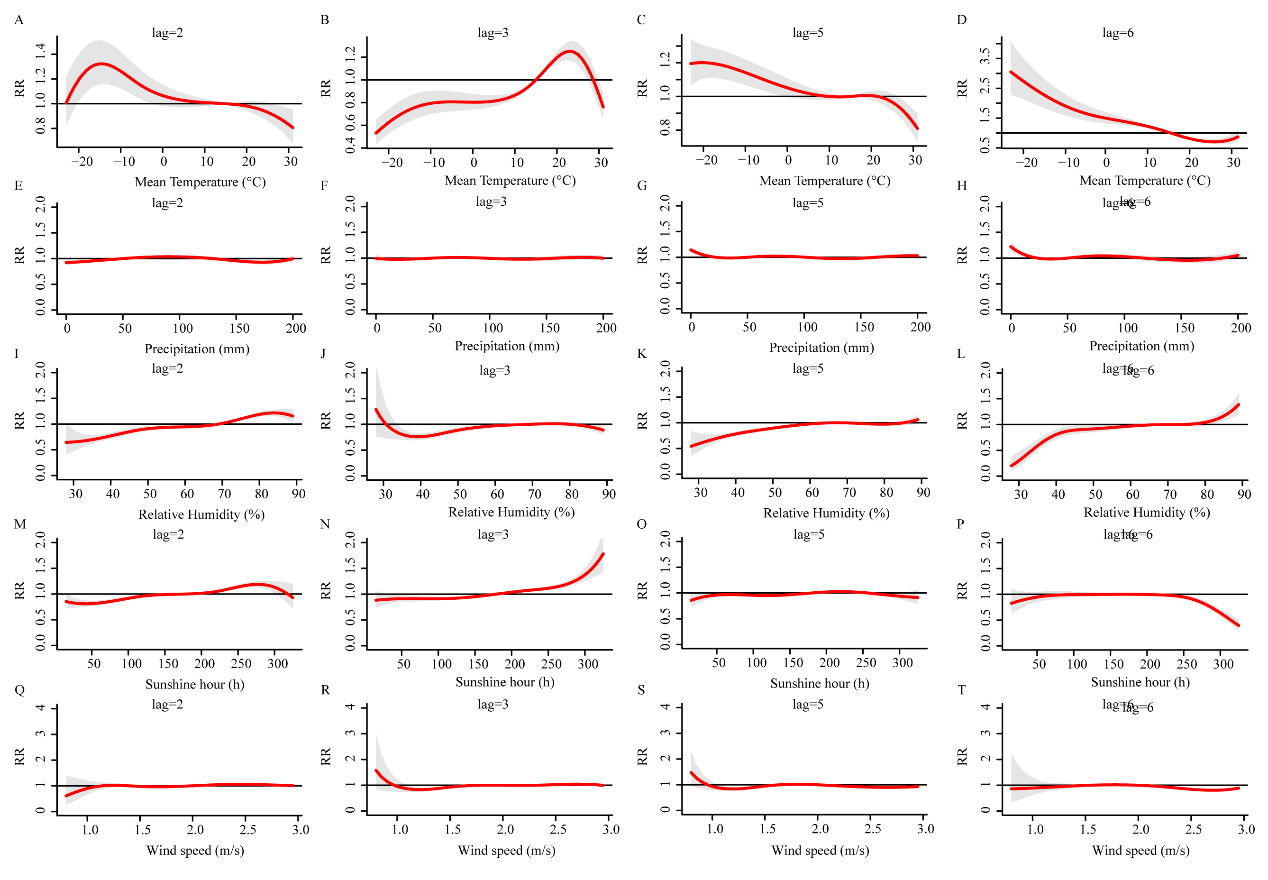


**Fig. S8: Geographical location of meteorological monitoring stations (2004-2018) in China**


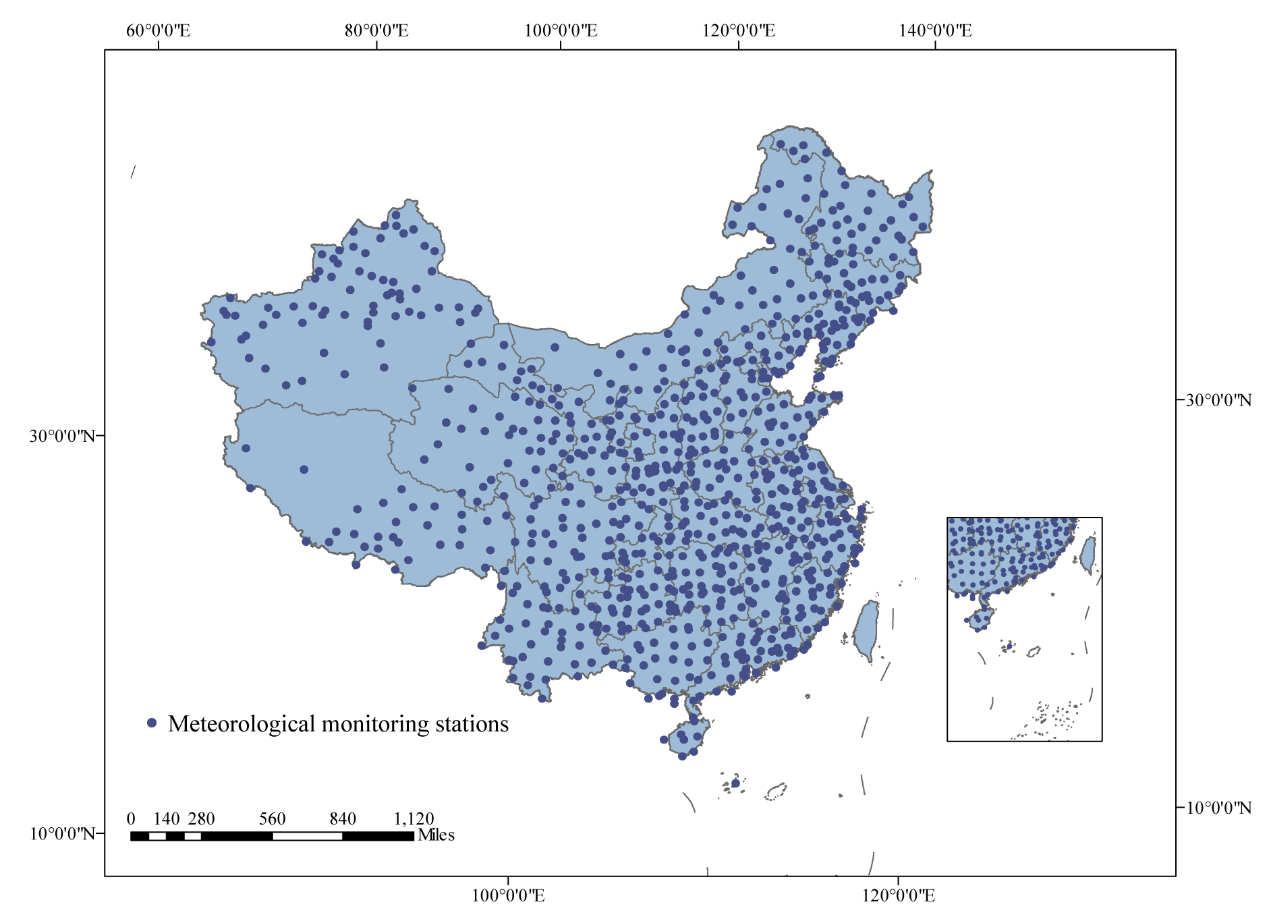
Meteorological monitoring stations (N=839). The map was created using ArcGIS 10.2 (Esri Inc, Redlands, CA, USA) (http://desktop.arcgis.com).

**Supplementary Data 1: National Incidence of HFRS (2004-1 to 2021-12)**

| Year | No. of cases | Population | Incidence (per 100000) |
| --- | --- | --- | --- |
| 2004-1 | 1743 | 1299880000 | 0.1341 |
| 2004-2 | 1688 | 1299880000 | 0.1299 |
| 2004-3 | 2226 | 1299880000 | 0.1712 |
| 2004-4 | 2446 | 1299880000 | 0.1882 |
| 2004-5 | 2636 | 1299880000 | 0.2028 |
| 2004-6 | 2097 | 1299880000 | 0.1613 |
| 2004-7 | 1352 | 1299880000 | 0.1040 |
| 2004-8 | 898 | 1299880000 | 0.0691 |
| 2004-9 | 983 | 1299880000 | 0.0756 |
| 2004-10 | 2417 | 1299880000 | 0.1859 |
| 2004-11 | 4013 | 1299880000 | 0.3087 |
| 2004-12 | 2542 | 1299880000 | 0.1956 |
| 2005-1 | 1864 | 1299879873 | 0.1434 |
| 2005-2 | 1379 | 1299879873 | 0.1061 |
| 2005-3 | 1888 | 1299879873 | 0.1452 |
| 2005-4 | 2076 | 1299879873 | 0.1597 |
| 2005-5 | 2287 | 1299879873 | 0.1759 |
| 2005-6 | 1922 | 1299879873 | 0.1479 |
| 2005-7 | 1174 | 1299879873 | 0.0903 |
| 2005-8 | 696 | 1299879873 | 0.0535 |
| 2005-9 | 685 | 1299879873 | 0.0527 |
| 2005-10 | 1768 | 1299879873 | 0.1360 |
| 2005-11 | 3255 | 1299879873 | 0.2504 |
| 2005-12 | 1883 | 1299879873 | 0.1449 |
| 2006-1 | 1442 | 1307559910 | 0.1103 |
| 2006-2 | 1201 | 1307559910 | 0.0919 |
| 2006-3 | 1446 | 1307559910 | 0.1106 |
| 2006-4 | 1397 | 1307559910 | 0.1068 |
| 2006-5 | 1526 | 1307559910 | 0.1167 |
| 2006-6 | 1508 | 1307559910 | 0.1153 |
| 2006-7 | 800 | 1307559910 | 0.0612 |
| 2006-8 | 531 | 1307559910 | 0.0406 |
| 2006-9 | 575 | 1307559910 | 0.0440 |
| 2006-10 | 1186 | 1307559910 | 0.0907 |
| 2006-11 | 2088 | 1307559910 | 0.1597 |
| 2006-12 | 1398 | 1307559910 | 0.1069 |
| 2007-1 | 918 | 1314476400 | 0.0698 |
| 2007-2 | 623 | 1314476400 | 0.0474 |
| 2007-3 | 724 | 1314476400 | 0.0551 |
| 2007-4 | 814 | 1314476400 | 0.0619 |
| 2007-5 | 990 | 1314476400 | 0.0753 |
| 2007-6 | 1172 | 1314476400 | 0.0892 |
| 2007-7 | 700 | 1314476400 | 0.0533 |
| 2007-8 | 489 | 1314476400 | 0.0372 |
| 2007-9 | 427 | 1314476400 | 0.0325 |
| 2007-10 | 1042 | 1314476400 | 0.0793 |
| 2007-11 | 1890 | 1314476400 | 0.1438 |
| 2007-12 | 1274 | 1314476400 | 0.0969 |
| 2008-1 | 675 | 1321290000 | 0.0511 |
| 2008-2 | 467 | 1321290000 | 0.0353 |
| 2008-3 | 539 | 1321290000 | 0.0408 |
| 2008-4 | 583 | 1321290000 | 0.0441 |
| 2008-5 | 768 | 1321290000 | 0.0581 |
| 2008-6 | 925 | 1321290000 | 0.0700 |
| 2008-7 | 536 | 1321290000 | 0.0406 |
| 2008-8 | 365 | 1321290000 | 0.0276 |
| 2008-9 | 370 | 1321290000 | 0.0280 |
| 2008-10 | 964 | 1321290000 | 0.0730 |
| 2008-11 | 1626 | 1321290000 | 0.1231 |
| 2008-12 | 1221 | 1321290000 | 0.0924 |
| 2009-1 | 663 | 1328019999 | 0.0499 |
| 2009-2 | 562 | 1328019999 | 0.0423 |
| 2009-3 | 517 | 1328019999 | 0.0389 |
| 2009-4 | 621 | 1328019999 | 0.0468 |
| 2009-5 | 824 | 1328019999 | 0.0620 |
| 2009-6 | 939 | 1328019999 | 0.0707 |
| 2009-7 | 614 | 1328019999 | 0.0462 |
| 2009-8 | 419 | 1328019999 | 0.0316 |
| 2009-9 | 373 | 1328019999 | 0.0281 |
| 2009-10 | 898 | 1328019999 | 0.0676 |
| 2009-11 | 1485 | 1328019999 | 0.1118 |
| 2009-12 | 830 | 1328019999 | 0.0625 |
| 2010-1 | 511 | 1334740003 | 0.0383 |
| 2010-2 | 329 | 1334740003 | 0.0246 |
| 2010-3 | 415 | 1334740003 | 0.0311 |
| 2010-4 | 503 | 1334740003 | 0.0377 |
| 2010-5 | 635 | 1334740003 | 0.0476 |
| 2010-6 | 724 | 1334740003 | 0.0542 |
| 2010-7 | 538 | 1334740003 | 0.0403 |
| 2010-8 | 316 | 1334740003 | 0.0237 |
| 2010-9 | 314 | 1334740003 | 0.0235 |
| 2010-10 | 1179 | 1334740003 | 0.0883 |
| 2010-11 | 2280 | 1334740003 | 0.1708 |
| 2010-12 | 1782 | 1334740003 | 0.1335 |
| 2011-1 | 886 | 1340909996 | 0.0661 |
| 2011-2 | 538 | 1340909996 | 0.0401 |
| 2011-3 | 546 | 1340909996 | 0.0407 |
| 2011-4 | 650 | 1340909996 | 0.0485 |
| 2011-5 | 865 | 1340909996 | 0.0645 |
| 2011-6 | 863 | 1340909996 | 0.0644 |
| 2011-7 | 588 | 1340909996 | 0.0439 |
| 2011-8 | 354 | 1340909996 | 0.0264 |
| 2011-9 | 367 | 1340909996 | 0.0274 |
| 2011-10 | 1212 | 1340909996 | 0.0904 |
| 2011-11 | 2139 | 1340909996 | 0.1595 |
| 2011-12 | 1771 | 1340909996 | 0.1321 |
| 2012-1 | 982 | 1347349996 | 0.0729 |
| 2012-2 | 675 | 1347349996 | 0.0501 |
| 2012-3 | 758 | 1347349996 | 0.0563 |
| 2012-4 | 766 | 1347349996 | 0.0569 |
| 2012-5 | 916 | 1347349996 | 0.0680 |
| 2012-6 | 1024 | 1347349996 | 0.0760 |
| 2012-7 | 600 | 1347349996 | 0.0445 |
| 2012-8 | 388 | 1347349996 | 0.0288 |
| 2012-9 | 405 | 1347349996 | 0.0301 |
| 2012-10 | 1566 | 1347349996 | 0.1162 |
| 2012-11 | 3002 | 1347349996 | 0.2228 |
| 2012-12 | 2226 | 1347349996 | 0.1652 |
| 2013-1 | 1279 | 1354040000 | 0.0945 |
| 2013-2 | 840 | 1354040000 | 0.0620 |
| 2013-3 | 939 | 1354040000 | 0.0693 |
| 2013-4 | 1031 | 1354040000 | 0.0761 |
| 2013-5 | 1261 | 1354040000 | 0.0931 |
| 2013-6 | 1249 | 1354040000 | 0.0922 |
| 2013-7 | 838 | 1354040000 | 0.0619 |
| 2013-8 | 523 | 1354040000 | 0.0386 |
| 2013-9 | 514 | 1354040000 | 0.0380 |
| 2013-10 | 1231 | 1354040000 | 0.0909 |
| 2013-11 | 1783 | 1354040000 | 0.1317 |
| 2013-12 | 1322 | 1354040000 | 0.0976 |
| 2014-1 | 928 | 1355168600 | 0.0685 |
| 2014-2 | 867 | 1355168600 | 0.0640 |
| 2014-3 | 983 | 1355168600 | 0.0725 |
| 2014-4 | 938 | 1355168600 | 0.0692 |
| 2014-5 | 1091 | 1355168600 | 0.0805 |
| 2014-6 | 1035 | 1355168600 | 0.0764 |
| 2014-7 | 682 | 1355168600 | 0.0503 |
| 2014-8 | 519 | 1355168600 | 0.0383 |
| 2014-9 | 497 | 1355168600 | 0.0367 |
| 2014-10 | 1039 | 1355168600 | 0.0767 |
| 2014-11 | 1732 | 1355168600 | 0.1278 |
| 2014-12 | 1211 | 1355168600 | 0.0894 |
| 2015-1 | 929 | 1362466686 | 0.0682 |
| 2015-2 | 631 | 1362466686 | 0.0463 |
| 2015-3 | 763 | 1362466686 | 0.0560 |
| 2015-4 | 781 | 1362466686 | 0.0573 |
| 2015-5 | 1039 | 1362466686 | 0.0763 |
| 2015-6 | 977 | 1362466686 | 0.0717 |
| 2015-7 | 634 | 1362466686 | 0.0465 |
| 2015-8 | 415 | 1362466686 | 0.0305 |
| 2015-9 | 445 | 1362466686 | 0.0327 |
| 2015-10 | 890 | 1362466686 | 0.0653 |
| 2015-11 | 1604 | 1362466686 | 0.1177 |
| 2015-12 | 1206 | 1362466686 | 0.0885 |
| 2016-1 | 887 | 1370784400 | 0.0647 |
| 2016-2 | 591 | 1370784400 | 0.0431 |
| 2016-3 | 705 | 1370784400 | 0.0514 |
| 2016-4 | 666 | 1370784400 | 0.0486 |
| 2016-5 | 842 | 1370784400 | 0.0614 |
| 2016-6 | 790 | 1370784400 | 0.0576 |
| 2016-7 | 515 | 1370784400 | 0.0376 |
| 2016-8 | 365 | 1370784400 | 0.0266 |
| 2016-9 | 390 | 1370784400 | 0.0285 |
| 2016-10 | 700 | 1370784400 | 0.0511 |
| 2016-11 | 1272 | 1370784400 | 0.0928 |
| 2016-12 | 1130 | 1370784400 | 0.0824 |
| 2017-1 | 709 | 1379837956 | 0.0514 |
| 2017-2 | 642 | 1379837956 | 0.0465 |
| 2017-3 | 734 | 1379837956 | 0.0532 |
| 2017-4 | 742 | 1379837956 | 0.0538 |
| 2017-5 | 1130 | 1379837956 | 0.0819 |
| 2017-6 | 1044 | 1379837956 | 0.0757 |
| 2017-7 | 676 | 1379837956 | 0.0490 |
| 2017-8 | 364 | 1379837956 | 0.0264 |
| 2017-9 | 345 | 1379837956 | 0.0250 |
| 2017-10 | 895 | 1379837956 | 0.0649 |
| 2017-11 | 2106 | 1379837956 | 0.1526 |
| 2017-12 | 1875 | 1379837956 | 0.1359 |
| 2018-1 | 984 | 1389096953 | 0.0708 |
| 2018-2 | 580 | 1389096953 | 0.0418 |
| 2018-3 | 833 | 1389096953 | 0.0600 |
| 2018-4 | 936 | 1389096953 | 0.0674 |
| 2018-5 | 1269 | 1389096953 | 0.0914 |
| 2018-6 | 1042 | 1389096953 | 0.0750 |
| 2018-7 | 714 | 1389096953 | 0.0514 |
| 2018-8 | 468 | 1389096953 | 0.0337 |
| 2018-9 | 353 | 1389096953 | 0.0254 |
| 2018-10 | 1099 | 1389096953 | 0.0791 |
| 2018-11 | 2174 | 1389096953 | 0.1565 |
| 2018-12 | 1514 | 1389096953 | 0.1090 |
| 2019-1 | 1109 | 1400050000 | 0.0792 |
| 2019-2 | 705 | 1400050000 | 0.0504 |
| 2019-3 | 738 | 1400050000 | 0.0527 |
| 2019-4 | 701 | 1400050000 | 0.0501 |
| 2019-5 | 939 | 1400050000 | 0.0671 |
| 2019-6 | 1007 | 1400050000 | 0.0719 |
| 2019-7 | 703 | 1400050000 | 0.0502 |
| 2019-8 | 411 | 1400050000 | 0.0294 |
| 2019-9 | 346 | 1400050000 | 0.0247 |
| 2019-10 | 649 | 1400050000 | 0.0464 |
| 2019-11 | 1445 | 1400050000 | 0.1032 |
| 2019-12 | 1364 | 1411780000 | 0.0966 |
| 2020-1 | 684 | 1411780000 | 0.0484 |
| 2020-2 | 374 | 1411780000 | 0.0265 |
| 2020-3 | 433 | 1411780000 | 0.0307 |
| 2020-4 | 540 | 1411780000 | 0.0382 |
| 2020-5 | 686 | 1411780000 | 0.0486 |
| 2020-6 | 826 | 1411780000 | 0.0585 |
| 2020-7 | 503 | 1411780000 | 0.0356 |
| 2020-8 | 313 | 1411780000 | 0.0222 |
| 2020-9 | 320 | 1411780000 | 0.0227 |
| 2020-10 | 611 | 1411780000 | 0.0433 |
| 2020-11 | 1796 | 1411780000 | 0.1272 |
| 2020-12 | 1460 | 1411780000 | 0.1034 |
| 2021-1 | 562 | 1443497378 | 0.0389 |
| 2021-2 | 365 | 1443497378 | 0.0253 |
| 2021-3 | 424 | 1443497378 | 0.0294 |
| 2021-4 | 510 | 1443497378 | 0.0353 |
| 2021-5 | 685 | 1443497378 | 0.0475 |
| 2021-6 | 724 | 1443497378 | 0.0502 |
| 2021-7 | 467 | 1443497378 | 0.0324 |
| 2021-8 | 255 | 1443497378 | 0.0177 |
| 2021-9 | 291 | 1443497378 | 0.0202 |
| 2021-10 | 678 | 1443497378 | 0.0470 |
| 2021-11 | 2120 | 1443497378 | 0.1469 |
| 2021-12 | 2402 | 1443497378 | 0.1664 |

**Supplementary Data 2: Incidence of HFRS in 31 provinces in China from 2004 to 2018.**

| **Province** | **2004** | **2005** | **2006** | **2007** | **2008** | **2009** | **2010** | **2011** | **2012** | **2013** | **2014** | **2015** | **2016** | **2017** | **2018** |
| --- | --- | --- | --- | --- | --- | --- | --- | --- | --- | --- | --- | --- | --- | --- | --- |
| Beijing | 0.952 | 0.703 | 0.410 | 0.158 | 0.104 | 0.071 | 0.085 | 0.107 | 0.089 | 0.130 | 0.071 | 0.056 | 0.041 | 0.032 | 0.037 |
| Tianjin | 2.271 | 1.239 | 0.518 | 0.279 | 0.170 | 0.162 | 0.130 | 0.100 | 0.207 | 0.219 | 0.258 | 0.211 | 0.200 | 0.205 | 0.128 |
| Hebei | 3.877 | 2.519 | 1.524 | 0.529 | 0.305 | 0.318 | 0.313 | 0.714 | 0.957 | 1.899 | 1.317 | 0.834 | 0.585 | 0.598 | 0.738 |
| Shanxi | 0.552 | 0.444 | 0.325 | 0.184 | 0.077 | 0.085 | 0.096 | 0.109 | 0.145 | 0.055 | 0.039 | 0.038 | 0.022 | 0.071 | 0.057 |
| Inner Mongolia | 1.845 | 1.966 | 1.077 | 0.551 | 0.337 | 0.584 | 0.376 | 0.324 | 0.290 | 0.454 | 0.829 | 0.659 | 0.422 | 0.369 | 0.593 |
| Liaoning | 13.046 | 9.993 | 4.646 | 2.309 | 1.715 | 1.669 | 1.743 | 2.242 | 2.569 | 2.964 | 3.000 | 2.179 | 1.969 | 2.462 | 2.584 |
| Jilin | 6.904 | 9.372 | 6.992 | 3.830 | 3.223 | 3.328 | 2.792 | 2.472 | 2.710 | 2.640 | 2.737 | 2.282 | 1.870 | 2.100 | 2.127 |
| Heilongjiang | 11.119 | 10.699 | 9.974 | 8.075 | 4.948 | 4.447 | 3.884 | 4.114 | 4.478 | 4.922 | 4.814 | 3.726 | 3.148 | 2.946 | 3.297 |
| Shanghai | 0.052 | 0.017 | 0.022 | 0.017 | 0.016 | 0.016 | 0.005 | 0.017 | 0.004 | 0.013 | 0.021 | 0.008 | 0.017 | 0.008 | 0.008 |
| Jiangsu | 0.562 | 0.373 | 0.371 | 0.356 | 0.320 | 0.361 | 0.381 | 0.291 | 0.320 | 0.441 | 0.442 | 0.278 | 0.414 | 0.469 | 0.336 |
| Zhejiang | 1.743 | 1.520 | 1.527 | 1.488 | 1.087 | 0.842 | 0.894 | 0.992 | 0.912 | 0.951 | 0.698 | 0.659 | 0.623 | 0.628 | 0.592 |
| Anhui | 0.362 | 0.298 | 0.317 | 0.268 | 0.172 | 0.181 | 0.220 | 0.225 | 0.317 | 0.322 | 0.264 | 0.261 | 0.337 | 0.441 | 0.459 |
| Fujian | 0.267 | 0.310 | 0.272 | 0.410 | 0.357 | 0.559 | 0.471 | 0.702 | 1.003 | 1.190 | 1.184 | 1.093 | 0.953 | 1.002 | 1.099 |
| Jiangxi | 0.852 | 1.031 | 0.916 | 0.864 | 0.794 | 0.775 | 0.893 | 1.227 | 1.352 | 1.534 | 1.143 | 1.543 | 1.481 | 1.243 | 1.525 |
| Shandong | 4.017 | 2.599 | 1.526 | 1.076 | 1.232 | 0.979 | 1.035 | 1.000 | 1.760 | 1.837 | 1.619 | 1.145 | 1.000 | 1.259 | 1.217 |
| Henan | 0.685 | 0.517 | 0.384 | 0.223 | 0.137 | 0.171 | 0.196 | 0.157 | 0.198 | 0.159 | 0.141 | 0.203 | 0.186 | 0.456 | 0.416 |
| Hubei | 0.594 | 0.378 | 0.499 | 0.400 | 0.286 | 0.438 | 0.395 | 0.493 | 0.389 | 0.440 | 0.410 | 0.438 | 0.403 | 0.858 | 1.564 |
| Hunan | 0.891 | 0.868 | 0.885 | 1.112 | 0.766 | 0.756 | 0.687 | 0.965 | 0.996 | 1.243 | 0.873 | 1.103 | 0.868 | 0.926 | 1.041 |
| Guangdong | 0.129 | 0.148 | 0.233 | 0.252 | 0.220 | 0.215 | 0.256 | 0.322 | 0.323 | 0.393 | 0.437 | 0.392 | 0.378 | 0.403 | 0.409 |
| Guangxi | 0.037 | 0.019 | 0.017 | 0.030 | 0.013 | 0.012 | 0.023 | 0.020 | 0.015 | 0.017 | 0.028 | 0.032 | 0.010 | 0.029 | 0.031 |
| Hainan | 0.000 | 0.000 | 0.000 | 0.000 | 0.000 | 0.000 | 0.000 | 0.012 | 0.000 | 0.023 | 0.045 | 0.033 | 0.033 | 0.033 | 0.050 |
| Chongqing | 0.112 | 0.118 | 0.057 | 0.057 | 0.071 | 0.056 | 0.031 | 0.045 | 0.041 | 0.054 | 0.071 | 0.020 | 0.043 | 0.016 | 0.039 |
| Sichuan | 0.520 | 0.343 | 0.162 | 0.140 | 0.122 | 0.077 | 0.079 | 0.073 | 0.060 | 0.061 | 0.083 | 0.124 | 0.134 | 0.315 | 0.351 |
| Guizhou | 0.521 | 0.327 | 0.257 | 0.136 | 0.199 | 0.222 | 0.247 | 0.271 | 0.271 | 0.321 | 0.237 | 0.131 | 0.167 | 0.163 | 0.128 |
| Yunnan | 0.126 | 0.100 | 0.088 | 0.031 | 0.042 | 0.033 | 0.033 | 0.041 | 0.106 | 0.212 | 0.525 | 0.560 | 0.456 | 0.480 | 0.527 |
| Tibet | 0.038 | 0.000 | 0.000 | 0.000 | 0.000 | 0.000 | 0.000 | 0.000 | 0.000 | 0.000 | 0.000 | 0.000 | 0.031 | 0.030 | 0.030 |
| Shaanxi | 4.934 | 3.806 | 2.761 | 2.771 | 3.767 | 3.732 | 6.384 | 6.979 | 9.595 | 3.709 | 2.760 | 3.706 | 2.460 | 5.413 | 4.544 |
| Gansu | 0.241 | 0.221 | 0.135 | 0.038 | 0.096 | 0.053 | 0.019 | 0.020 | 0.117 | 0.054 | 0.112 | 0.127 | 0.073 | 0.077 | 0.545 |
| Qinghai | 0.019 | 0.000 | 0.018 | 0.018 | 0.000 | 0.000 | 0.000 | 0.000 | 0.000 | 0.000 | 0.035 | 0.017 | 0.000 | 0.000 | 0.033 |
| Ningxia | 0.188 | 0.085 | 0.034 | 0.033 | 0.016 | 0.000 | 0.016 | 0.000 | 0.031 | 0.031 | 0.031 | 0.015 | 0.060 | 0.030 | 0.073 |
| Xinjiang | 0.000 | 0.000 | 0.000 | 0.000 | 0.005 | 0.009 | 0.000 | 0.005 | 0.005 | 0.000 | 0.004 | 0.000 | 0.004 | 0.013 | 0.004 |

**Supplementary Data 3: Meteorological factor data (839 urban areas in 31 provinces, with the data covering the period from 2004 through 2020)**

| Date | Mean temperature (°C) | | Precipitation (mm) | Relative humidity (%) | Sunlight (h) | Wind speed (m/s) |
| --- | --- | --- | --- | --- | --- | --- |
| 1/1/2004 | | -1.71 | 19.71 | 65.33 | 145.66 | 1.92 |
| 2/1/2004 | | 2.72 | 28.87 | 59.72 | 172.43 | 2.29 |
| 3/1/2004 | | 7.18 | 33.98 | 58.73 | 177.06 | 2.54 |
| 4/1/2004 | | 14.43 | 60.88 | 56.86 | 214.42 | 2.56 |
| 5/1/2004 | | 18.13 | 110.95 | 60.97 | 214.36 | 2.51 |
| 6/1/2004 | | 21.90 | 102.52 | 65.18 | 204.94 | 2.15 |
| 7/1/2004 | | 23.67 | 157.50 | 71.08 | 216.71 | 2.11 |
| 8/1/2004 | | 22.70 | 120.54 | 73.46 | 204.01 | 1.97 |
| 9/1/2004 | | 18.99 | 81.40 | 70.57 | 191.00 | 1.94 |
| 10/1/2004 | | 12.64 | 19.38 | 63.71 | 200.40 | 1.92 |
| 11/1/2004 | | 6.75 | 32.18 | 66.74 | 168.02 | 1.95 |
| 12/1/2004 | | 0.52 | 20.98 | 68.19 | 148.07 | 2.03 |
| 1/1/2005 | | -2.35 | 17.63 | 65.68 | 140.67 | 1.95 |
| 2/1/2005 | | -0.89 | 39.36 | 65.69 | 125.14 | 2.28 |
| 3/1/2005 | | 6.06 | 40.27 | 58.34 | 184.52 | 2.44 |
| 4/1/2005 | | 14.16 | 50.12 | 56.21 | 208.69 | 2.59 |
| 5/1/2005 | | 18.29 | 121.81 | 62.83 | 205.05 | 2.45 |
| 6/1/2005 | | 22.83 | 144.38 | 65.51 | 203.48 | 2.17 |
| 7/1/2005 | | 24.39 | 136.93 | 70.44 | 208.29 | 2.14 |
| 8/1/2005 | | 22.71 | 142.64 | 73.83 | 184.45 | 2.00 |
| 9/1/2005 | | 19.96 | 84.44 | 69.86 | 190.01 | 1.96 |
| 10/1/2005 | | 13.23 | 42.19 | 65.91 | 180.82 | 2.04 |
| 11/1/2005 | | 7.36 | 26.83 | 65.09 | 157.79 | 1.92 |
| 12/1/2005 | | -1.42 | 12.24 | 59.93 | 157.09 | 2.05 |
| 1/1/2006 | | -1.31 | 20.35 | 67.78 | 131.49 | 2.02 |
| 2/1/2006 | | 1.35 | 38.04 | 66.15 | 129.09 | 2.36 |
| 3/1/2006 | | 7.04 | 34.94 | 57.14 | 187.97 | 2.42 |
| 4/1/2006 | | 13.38 | 67.23 | 57.59 | 193.16 | 2.64 |
| 5/1/2006 | | 18.40 | 115.36 | 59.86 | 213.29 | 2.46 |
| 6/1/2006 | | 22.23 | 137.60 | 66.38 | 196.80 | 2.17 |
| 7/1/2006 | | 24.44 | 151.90 | 71.06 | 207.38 | 2.14 |
| 8/1/2006 | | 23.94 | 106.09 | 70.26 | 218.05 | 1.94 |
| 9/1/2006 | | 18.78 | 65.47 | 68.44 | 190.33 | 1.91 |
| 10/1/2006 | | 14.92 | 39.09 | 67.74 | 180.25 | 1.81 |
| 11/1/2006 | | 7.30 | 40.27 | 66.90 | 153.96 | 1.97 |
| 12/1/2006 | | 0.37 | 12.43 | 66.96 | 148.51 | 1.86 |
| 1/1/2007 | | -1.14 | 18.75 | 63.56 | 152.83 | 2.10 |
| 2/1/2007 | | 4.32 | 28.87 | 62.56 | 157.65 | 2.12 |
| 3/1/2007 | | 7.20 | 48.82 | 63.54 | 167.12 | 2.12 |
| 4/1/2007 | | 13.01 | 63.87 | 57.18 | 193.79 | 2.11 |
| 5/1/2007 | | 19.42 | 78.21 | 57.31 | 228.01 | 2.11 |
| 6/1/2007 | | 22.42 | 135.03 | 65.96 | 190.74 | 2.09 |
| 7/1/2007 | | 23.99 | 147.23 | 70.52 | 207.26 | 2.07 |
| 8/1/2007 | | 23.61 | 145.27 | 71.55 | 206.02 | 2.09 |
| 9/1/2007 | | 19.06 | 83.49 | 70.19 | 175.51 | 2.09 |
| 10/1/2007 | | 13.14 | 43.27 | 67.21 | 165.53 | 2.09 |
| 11/1/2007 | | 6.35 | 11.37 | 61.95 | 179.09 | 2.11 |
| 12/1/2007 | | 1.33 | 17.54 | 67.28 | 131.38 | 2.10 |
| 1/1/2008 | | -3.32 | 28.65 | 65.70 | 125.91 | 2.06 |
| 2/1/2008 | | -1.17 | 20.02 | 60.30 | 164.89 | 2.11 |
| 3/1/2008 | | 8.67 | 40.22 | 58.81 | 187.71 | 2.25 |
| 4/1/2008 | | 13.92 | 63.57 | 59.21 | 181.96 | 2.44 |
| 5/1/2008 | | 18.65 | 93.82 | 59.75 | 211.57 | 2.32 |
| 6/1/2008 | | 21.80 | 155.69 | 67.47 | 181.91 | 2.10 |
| 7/1/2008 | | 24.06 | 157.39 | 71.03 | 205.53 | 2.07 |
| 8/1/2008 | | 22.81 | 135.62 | 71.96 | 198.52 | 1.93 |
| 9/1/2008 | | 19.48 | 74.65 | 70.51 | 185.50 | 1.97 |
| 10/1/2008 | | 13.94 | 59.91 | 68.56 | 173.65 | 1.87 |
| 11/1/2008 | | 6.27 | 38.28 | 64.47 | 168.51 | 1.95 |
| 12/1/2008 | | 0.61 | 8.11 | 59.40 | 162.39 | 2.10 |
| 1/1/2009 | | -1.71 | 10.16 | 59.59 | 161.10 | 1.99 |
| 2/1/2009 | | 3.62 | 25.51 | 63.18 | 137.54 | 2.22 |
| 3/1/2009 | | 7.00 | 49.56 | 59.51 | 177.52 | 2.44 |
| 4/1/2009 | | 14.05 | 70.07 | 58.10 | 193.62 | 2.39 |
| 5/1/2009 | | 18.51 | 89.53 | 59.09 | 212.31 | 2.37 |
| 6/1/2009 | | 22.23 | 127.93 | 64.05 | 207.05 | 2.20 |
| 7/1/2009 | | 23.99 | 138.25 | 69.75 | 207.09 | 2.06 |
| 8/1/2009 | | 23.12 | 121.59 | 70.83 | 207.40 | 1.97 |
| 9/1/2009 | | 19.62 | 66.68 | 70.04 | 183.93 | 1.96 |
| 10/1/2009 | | 14.20 | 30.52 | 63.67 | 192.24 | 1.88 |
| 11/1/2009 | | 4.15 | 41.98 | 67.00 | 153.35 | 2.17 |
| 12/1/2009 | | -0.49 | 20.57 | 66.50 | 141.08 | 1.95 |
| 1/1/2010 | | -1.07 | 22.78 | 63.25 | 146.99 | 2.05 |
| 2/1/2010 | | 1.37 | 30.34 | 64.07 | 136.47 | 2.25 |
| 3/1/2010 | | 5.96 | 52.74 | 59.70 | 171.03 | 2.61 |
| 4/1/2010 | | 11.05 | 87.62 | 60.52 | 175.05 | 2.56 |
| 5/1/2010 | | 18.19 | 113.53 | 63.89 | 190.97 | 2.37 |
| 6/1/2010 | | 22.04 | 134.57 | 67.34 | 188.41 | 2.05 |
| 7/1/2010 | | 24.70 | 158.37 | 71.53 | 202.12 | 2.03 |
| 8/1/2010 | | 23.57 | 133.03 | 71.04 | 218.16 | 1.93 |
| 9/1/2010 | | 19.59 | 108.78 | 73.06 | 173.34 | 1.86 |
| 10/1/2010 | | 12.73 | 56.96 | 68.91 | 172.70 | 1.97 |
| 11/1/2010 | | 6.71 | 16.32 | 64.19 | 176.23 | 1.94 |
| 12/1/2010 | | 0.06 | 31.89 | 59.95 | 169.40 | 2.20 |
| 1/1/2011 | | -5.05 | 11.97 | 59.58 | 145.06 | 2.06 |
| 2/1/2011 | | 1.72 | 17.36 | 62.31 | 145.01 | 2.08 |
| 3/1/2011 | | 5.11 | 27.03 | 55.57 | 189.43 | 2.36 |
| 4/1/2011 | | 13.57 | 29.70 | 54.42 | 211.70 | 2.45 |
| 5/1/2011 | | 18.06 | 86.10 | 59.20 | 219.59 | 2.39 |
| 6/1/2011 | | 22.37 | 148.23 | 66.90 | 191.43 | 2.15 |
| 7/1/2011 | | 24.18 | 122.17 | 69.55 | 212.58 | 2.00 |
| 8/1/2011 | | 23.37 | 112.56 | 70.87 | 220.42 | 1.89 |
| 9/1/2011 | | 18.56 | 84.85 | 69.18 | 176.66 | 1.95 |
| 10/1/2011 | | 13.27 | 54.99 | 68.26 | 169.31 | 1.91 |
| 11/1/2011 | | 7.74 | 46.23 | 71.23 | 145.40 | 1.84 |
| 12/1/2011 | | -0.61 | 10.47 | 64.48 | 147.30 | 1.87 |
| 1/1/2012 | | -3.38 | 29.02 | 67.37 | 126.55 | 1.84 |
| 2/1/2012 | | -0.43 | 25.86 | 60.73 | 135.17 | 2.19 |
| 3/1/2012 | | 5.99 | 52.20 | 61.54 | 167.95 | 2.34 |
| 4/1/2012 | | 14.14 | 77.76 | 57.23 | 199.24 | 2.50 |
| 5/1/2012 | | 19.06 | 104.17 | 61.64 | 210.12 | 2.21 |
| 6/1/2012 | | 22.04 | 138.53 | 68.88 | 176.20 | 2.05 |
| 7/1/2012 | | 24.29 | 161.90 | 72.25 | 207.77 | 2.04 |
| 8/1/2012 | | 23.25 | 124.06 | 71.53 | 212.61 | 1.97 |
| 9/1/2012 | | 18.53 | 91.46 | 70.45 | 185.69 | 1.83 |
| 10/1/2012 | | 13.07 | 40.74 | 65.15 | 188.53 | 1.86 |
| 11/1/2012 | | 5.21 | 53.85 | 66.22 | 147.06 | 2.04 |
| 12/1/2012 | | -1.45 | 30.90 | 65.88 | 132.94 | 2.10 |
| 1/1/2013 | | -2.21 | 9.29 | 65.03 | 149.59 | 1.84 |
| 2/1/2013 | | 1.57 | 22.92 | 64.81 | 135.58 | 2.17 |
| 3/1/2013 | | 8.26 | 45.33 | 56.38 | 199.16 | 2.45 |
| 4/1/2013 | | 12.61 | 66.38 | 57.25 | 199.48 | 2.50 |
| 5/1/2013 | | 18.87 | 119.77 | 63.02 | 201.18 | 2.32 |
| 6/1/2013 | | 22.40 | 123.39 | 67.47 | 202.92 | 2.21 |
| 7/1/2013 | | 24.49 | 162.83 | 71.16 | 214.37 | 2.15 |
| 8/1/2013 | | 24.17 | 129.31 | 69.43 | 227.23 | 2.10 |
| 9/1/2013 | | 18.97 | 86.93 | 69.65 | 185.81 | 1.95 |
| 10/1/2013 | | 13.44 | 39.97 | 64.79 | 194.51 | 1.91 |
| 11/1/2013 | | 6.62 | 36.20 | 64.89 | 162.40 | 2.03 |
| 12/1/2013 | | -0.11 | 26.83 | 62.29 | 165.69 | 1.82 |
| 1/1/2014 | | -0.28 | 11.56 | 58.32 | 176.90 | 1.99 |
| 2/1/2014 | | 0.35 | 33.92 | 65.52 | 123.31 | 2.17 |
| 3/1/2014 | | 7.95 | 170.78 | 59.45 | 183.39 | 2.23 |
| 4/1/2014 | | 14.20 | 71.19 | 60.40 | 181.76 | 2.34 |
| 5/1/2014 | | 18.30 | 122.64 | 60.78 | 200.89 | 2.44 |
| 6/1/2014 | | 22.07 | 135.61 | 68.87 | 177.20 | 2.05 |
| 7/1/2014 | | 24.11 | 149.29 | 70.71 | 220.65 | 2.08 |
| 8/1/2014 | | 22.54 | 136.24 | 73.03 | 194.84 | 1.87 |
| 9/1/2014 | | 19.30 | 107.59 | 72.99 | 175.72 | 1.93 |
| 10/1/2014 | | 13.95 | 41.39 | 66.65 | 192.69 | 2.00 |
| 11/1/2014 | | 6.93 | 36.31 | 67.33 | 143.18 | 2.00 |
| 12/1/2014 | | -0.63 | 19.34 | 59.10 | 163.36 | 2.15 |
| 1/1/2015 | | -0.08 | 22.41 | 63.70 | 149.97 | 2.01 |
| 2/1/2015 | | 2.41 | 26.52 | 62.38 | 145.74 | 2.19 |
| 3/1/2015 | | 7.92 | 35.64 | 59.42 | 176.44 | 2.41 |
| 4/1/2015 | | 13.60 | 61.44 | 59.37 | 200.86 | 2.55 |
| 5/1/2015 | | 18.67 | 128.67 | 62.76 | 202.97 | 2.48 |
| 6/1/2015 | | 22.03 | 148.42 | 68.89 | 184.51 | 2.29 |
| 7/1/2015 | | 23.57 | 191.04 | 69.32 | 212.33 | 2.10 |
| 8/1/2015 | | 22.97 | 169.92 | 71.66 | 205.31 | 1.98 |
| 9/1/2015 | | 19.02 | 95.01 | 73.37 | 168.22 | 1.94 |
| 10/1/2015 | | 13.64 | 55.97 | 65.89 | 188.96 | 2.08 |
| 11/1/2015 | | 6.80 | 182.60 | 74.23 | 114.99 | 1.99 |
| 12/1/2015 | | 0.87 | 161.89 | 70.22 | 124.71 | 1.97 |
| 1/1/2016 | | -2.29 | 55.32 | 66.47 | 135.96 | 2.14 |
| 2/1/2016 | | 1.10 | 24.06 | 58.64 | 175.05 | 2.30 |
| 3/1/2016 | | 8.02 | 48.97 | 58.97 | 180.94 | 2.40 |
| 4/1/2016 | | 14.50 | 99.51 | 62.09 | 177.78 | 2.49 |
| 5/1/2016 | | 18.16 | 120.34 | 63.12 | 198.72 | 2.55 |
| 6/1/2016 | | 22.30 | 155.88 | 68.69 | 202.21 | 2.24 |
| 7/1/2016 | | 24.61 | 163.79 | 72.27 | 215.07 | 2.16 |
| 8/1/2016 | | 24.02 | 120.64 | 72.27 | 216.42 | 2.05 |
| 9/1/2016 | | 19.77 | 99.55 | 71.86 | 173.10 | 1.95 |
| 10/1/2016 | | 13.47 | 82.85 | 72.23 | 142.55 | 2.16 |
| 11/1/2016 | | 5.86 | 46.34 | 69.56 | 138.85 | 2.11 |
| 12/1/2016 | | 2.11 | 21.39 | 68.21 | 145.83 | 1.99 |
| 1/1/2017 | | 0.05 | 19.99 | 66.05 | 182.31 | 2.05 |
| 2/1/2017 | | 2.51 | 21.37 | 61.68 | 165.93 | 2.28 |
| 3/1/2017 | | 6.84 | 59.54 | 62.52 | 183.99 | 2.26 |
| 4/1/2017 | | 14.08 | 61.95 | 59.02 | 179.15 | 2.53 |
| 5/1/2017 | | 19.00 | 80.29 | 58.90 | 183.71 | 2.48 |
| 6/1/2017 | | 21.83 | 172.04 | 68.27 | 177.29 | 2.18 |
| 7/1/2017 | | 25.07 | 138.24 | 69.94 | 182.76 | 2.17 |
| 8/1/2017 | | 23.52 | 192.88 | 73.83 | 181.26 | 2.08 |
| 9/1/2017 | | 19.92 | 87.91 | 71.91 | 176.82 | 2.00 |
| 10/1/2017 | | 13.03 | 68.04 | 70.67 | 181.69 | 2.13 |
| 11/1/2017 | | 6.32 | 26.49 | 64.08 | 175.36 | 2.16 |
| 12/1/2017 | | 0.46 | 8.24 | 60.32 | 181.96 | 2.04 |
| 1/1/2018 | | -2.24 | 37.09 | 65.08 | 134.06 | 2.22 |
| 2/1/2018 | | 0.58 | 13.68 | 58.16 | 153.90 | 2.26 |
| 3/1/2018 | | 9.03 | 46.53 | 61.68 | 187.37 | 2.44 |
| 4/1/2018 | | 14.54 | 61.97 | 58.78 | 194.29 | 2.65 |
| 5/1/2018 | | 19.43 | 106.89 | 63.73 | 205.76 | 2.46 |
| 6/1/2018 | | 22.67 | 131.09 | 68.07 | 190.14 | 2.20 |
| 7/1/2018 | | 25.06 | 153.21 | 75.06 | 201.69 | 2.20 |
| 8/1/2018 | | 24.19 | 164.39 | 75.63 | 204.10 | 2.03 |
| 9/1/2018 | | 19.20 | 96.08 | 72.52 | 170.77 | 2.05 |
| 10/1/2018 | | 12.63 | 37.86 | 65.53 | 183.77 | 2.00 |
| 11/1/2018 | | 6.62 | 41.84 | 69.98 | 143.82 | 1.98 |
| 12/1/2018 | | -0.15 | 30.73 | 65.58 | 121.47 | 2.22 |
| 1/1/2019 | | -0.81 | 21.95 | 64.74 | 122.16 | 2.05 |
| 2/1/2019 | | 1.38 | 37.80 | 64.46 | 108.30 | 2.25 |
| 3/1/2019 | | 8.02 | 51.93 | 58.08 | 175.79 | 2.37 |
| 4/1/2019 | | 14.48 | 75.40 | 60.16 | 171.19 | 2.53 |
| 5/1/2019 | | 18.21 | 94.72 | 60.00 | 179.41 | 2.55 |
| 6/1/2019 | | 22.24 | 144.24 | 68.08 | 151.90 | 2.25 |
| 7/1/2019 | | 24.06 | 152.51 | 72.48 | 181.14 | 2.08 |
| 8/1/2019 | | 23.72 | 137.71 | 71.74 | 187.67 | 2.09 |
| 9/1/2019 | | 19.94 | 67.12 | 68.30 | 162.94 | 2.03 |
| 10/1/2019 | | 13.72 | 47.98 | 67.93 | 176.54 | 2.07 |
| 11/1/2019 | | 6.99 | 18.31 | 63.90 | 153.40 | 2.18 |
| 12/1/2019 | | 0.71 | 17.64 | 66.62 | 153.78 | 2.01 |
| 1/1/2020 | | -0.28 | 38.77 | 71.29 | 144.42 | 1.93 |
| 2/1/2020 | | 2.97 | 35.75 | 65.11 | 169.34 | 2.18 |
| 3/1/2020 | | 8.51 | 58.12 | 60.54 | 195.41 | 2.49 |
| 4/1/2020 | | 12.82 | 45.48 | 54.96 | 219.00 | 2.48 |
| 5/1/2020 | | 19.23 | 97.14 | 61.53 | 226.53 | 2.51 |
| 6/1/2020 | | 22.58 | 161.18 | 68.23 | 200.18 | 2.36 |
| 7/1/2020 | | 23.83 | 161.76 | 73.15 | 199.34 | 2.15 |
| 8/1/2020 | | 23.71 | 159.17 | 74.18 | 214.15 | 2.13 |
| 9/1/2020 | | 19.11 | 119.98 | 74.57 | 165.87 | 1.96 |
| 10/1/2020 | | 12.70 | 44.49 | 67.11 | 169.04 | 2.14 |
| 11/1/2020 | | 7.11 | 25.18 | 66.36 | 164.25 | 2.18 |
| 12/1/2020 | | -0.80 | 9.25 | 65.33 | 152.47 | 2.12 |
